# Fission yeast Ccq1 is a modulator of telomerase activity

**DOI:** 10.1093/nar/gkx1223

**Published:** 2017-12-04

**Authors:** Christine A Armstrong, Vera Moiseeva, Laura C Collopy, Siân R Pearson, Tomalika R Ullah, Shidong T Xi, Jennifer Martin, Shaan Subramaniam, Sara Marelli, Hanna Amelina, Kazunori Tomita

**Affiliations:** 1 Chromosome Maintenance Group, UCL Cancer Institute, University College London, London WC1E 6DD, UK; 2 MSc Human Molecular Genetics, Faculty of Medicine, Imperial College London, London SW7 2AZ, UK; 3 Faculty of Life Sciences, University College London, London WC1E 6BT, UK

## Abstract

Shelterin, the telomeric protein complex, plays a crucial role in telomere homeostasis. In fission yeast, telomerase is recruited to chromosome ends by the shelterin component Tpz1 and its binding partner Ccq1, where telomerase binds to the 3′ overhang to add telomeric repeats. Recruitment is initiated by the interaction of Ccq1 with the telomerase subunit Est1. However, how telomerase is released following elongation remains to be established. Here, we show that Ccq1 also has a role in the *suppression* of telomere elongation, when coupled with the Clr4 histone H3 methyl-transferase complex and the Clr3 histone deacetylase and nucleosome remodelling complex, SHREC. We have dissected the functions of Ccq1 by establishing a Ccq1-Est1 fusion system, which bypasses the telomerase recruitment step. We demonstrate that Ccq1 forms two distinct complexes for positive and negative telomerase regulation, with Est1 and Clr3 respectively. The negative form of Ccq1 promotes dissociation of Ccq1-telomerase from Tpz1, thereby restricting local telomerase activity. The Clr4 complex also has a negative regulation activity with Ccq1, independently of SHREC. Thus, we propose a model in which Ccq1-Est1 recruits telomerase to mediate telomere extension, whilst elongated telomeric DNA recruits Ccq1 with the chromatin-remodelling complexes, which in turn releases telomerase from the telomere.

## INTRODUCTION

Through each round of cell division, telomeres get progressively shorter, eventually triggering replicative senescence. Therefore, telomere length largely defines the proliferative capacity of the cell. Stem cells and unicellular eukaryotic organisms make use of the ribonucleoprotein complex telomerase to replenish eroded telomeres and evade replicative senescence. Telomerase activity is tightly controlled at multiple stages (1,2). The active telomerase complex is recruited preferentially to short telomeres only during S-phase (3–9). This recruitment process requires interaction between telomerase and the telomeric protein complex, shelterin (10–20). In humans, once telomerase is stably docked at the tip of the telomere and has gained access to the 3′ overhang, telomerase adds telomeric repeats in a processive manner (17,21). The rate of this processivity, in addition to the efficiency and frequency of telomerase recruitment, counteracts shortening of telomeres and therefore defines telomere length homeostasis (22) (reviewed in (2)).

The shelterin complex is a conserved group of proteins that plays a number of crucial roles in maintaining telomere homeostasis (23). Shelterin aids the compaction of telomeric DNA into heterochromatin to effectively shield chromosome ends from inappropriate activation of the DNA damage response machineries (24). Access of telomerase to the telomeres is therefore thought to be tightly controlled by modifications to the shelterin proteins. In fission yeast, shelterin contains the telomeric double-stranded DNA (dsDNA) binding protein Taz1 and the single-stranded DNA (ssDNA) binding protein, Pot1. Taz1 and Pot1 are bridged by the connecting proteins Rap1, Poz1 and Tpz1 (25). In turn, Tpz1 also complexes with the telomeric protein Ccq1 for telomerase recruitment and checkpoint suppression (25,26). The linkage between the telomeric ds- and ssDNA is important, as disruption of these protein-protein interactions leads to hyper activation of telomerase at telomeres (27,28).

To initiate telomere lengthening, telomerase must be recruited to the telomeres and translocated to the single-stranded 3′ end. Once there, in fission yeast the reverse transcriptase component, Trt1, can add telomeric DNA repeats from an RNA template, provided by the RNA component, TER1. Telomerase recruitment is initiated by phosphorylation of threonine 93 in Ccq1, which promotes the interaction of Ccq1 with the TER1 binding protein, Est1 (13,18,29). This interaction is thought to temporarily release Ccq1 from Tpz1 and Est1 from TER1. Further direct interaction between Tpz1 and Trt1 and re-association of Ccq1 to Tpz1 are necessary to initiate telomerase activity (11,30). A similar process of telomerase activation has been proposed for budding yeast and human cells (16,31). In human cells, telomerase directly binds to the Tpz1 equivalent ACD (previously called TPP1) and this interaction stabilises telomerase at the telomere and promotes its processivity (14,17,19,20). Thus, the shelterin components Tpz1 and ACD^TPP1^ are crucial for both stable association of telomerase and processivity.

During late S/G2 phase, the Stn1–Ten1 complex is recruited to the telomeres to terminate further telomerase recruitment (32). In fission yeast, this is achieved by SUMOylation of Tpz1 at the position of lysine 242 (33,34). However, it remains unknown how retention of telomerase is monitored and regulated to meet the needs of the telomeres during S-phase. In human cancer cells, telomerase can probe telomeres and be repeatedly recruited to the telomere for extension during S-phase (35). In this regard, mechanisms that promote temporal telomerase dissociation are to be anticipated.

Ccq1 is a structural homolog of budding yeast HDA2/3, an essential component for HDA1 histone deacetylase (HDAC) (36). Ccq1 is known to associate with the nucleosome remodelling and deacetylation complex, SHREC (Snf2/HDAC-containing repressor complex), composed of Clr1, Clr2, Clr3 and Mit1. This occurs *via* a direct interaction with Clr3 (37,38). Clr3 suppresses local transcription by RNA polymerase II at heterochromatic regions (39). Ccq1-associated SHREC binds at the telomere *via* shelterin, and also at the subtelomeric region *via* Swi6 (a fission yeast heterochromatin protein 1, HP1) (37,40–42). Ccq1-SHREC also binds to selected genes encoding non-coding RNA (37). Ccq1 additionally interacts with the Clr4 complex (a cullin-ring finger-ligase-4 complex, or CRL4 in humans), which has Histone H3 methyltransferase and ubiquitin ligase activity (43,44), to establish heterochromatin formation along telomeric DNA repeats (45). Thus, Ccq1 appears to hold two distinct functions; telomerase recruitment and activation, in addition to heterochromatic gene silencing. Whether the chromatin remodelling function of Ccq1 is involved in telomere length homeostasis remains to be investigated.

Chromatin compaction is indeed associated with transcriptional repression. Recent studies, however, revealed that subtelomeric and telomeric non-coding DNA are actually transcribed (46). One of the telomeric non-coding RNAs, the telomeric repeat containing RNA (TERRA) is known to control telomere length homeostasis. Like gene transcription, TERRA is also transcribed by RNA polymerase II (47). In fission yeast, the shelterin proteins Taz1 and Rap1 repress TERRA expression, and the level of expression is therefore correlated with the length of telomeres (47,48). Although the fundamental function of TERRA remains to be established, in fission yeast TERRA is likely to trap telomerase at the telomere and therefore promote local telomerase activity (49). Thus, regulation of TERRA transcription is crucial for telomere length homeostasis.

To investigate the downstream processes of telomerase activity in fission yeast, we made use of an engineered protein comprising Ccq1 fused to Est1, which bypasses the recruitment step. This allowed us to study whether Ccq1 has a role other than telomerase recruitment. Here, we report that Ccq1 also serves as a negative regulator of telomerase activity, promoted *via* its C-terminal region. The interactions of Ccq1 with Est1 and Clr3-SHREC appear to be mutually exclusive, thus functionally separating Ccq1 as a positive and negative regulator of telomere length. Ccq1 also functions independently with Clr4 to negatively regulate telomere length. We propose a model in which elongated telomeres recruit negative-regulatory forms of Ccq1 and release telomerase-associated Ccq1. Thus, Ccq1 is a key modulator of both positive and negative telomerase activity.

## MATERIALS AND METHODS

### Strain construction and culture

Fission yeast strains used for this study are listed in Supplementary Table S1. Fission yeasts were cultured in standard rich medium (YES: FORMEDIUM™) at 32°C unless indicated. For the gene silencing assay and live cell imaging, cells were cultured in synthetic minimal media (EMM: FORMEDIUM™).

Truncations and mutations of the *ccq1* gene were generated by replacement of the endogenous gene with ccq1 C-tag targeting plasmids. The *ccq1* gene including 800 bases of the upstream region was cloned into the C-terminal tagging pNX3 vectors (pNX3c-FL3, pNX3b-PK3, pNX3c-Myc13 and pNX3a-FL3YFP: (50)). The downstream 370 bases of the ccq1 gene was also cloned with the addition of an AflII site and inserted after the selection marker to generate the *ccq1* C-terminal tagging plasmid. The cloned *ccq1* gene from the plasmid was mutated and truncated using PCR-based methods and the QuickChange Lightning site-directed mutagenesis kit (Agilent Technologies). The ccq1 plasmid was digested with *Afl*II to generate the *ccq1* targeting fragment. A plasmid for Ccq1 fused Est1 was constructed by cloning the *ccq1* gene and inserting it before the *13xMyc* sequence of the est1 N-terminal 13xMyc tagging plasmid, pEst1b-nMyc13, described previously (11). All other strains were generated by genetic crossing or PCR-based gene targeting (50).

### Southern blot

Genomic DNA for telomere Southern blot was prepared from strains cultured more than two weeks after generation, unless indicated. Genomic DNA was quantified and normalised, and digested with EcoRI. The digested DNA fragments were separated on 1% agarose gel and subjected to Southern blotting with a 500 base telomere probe as described before (11).

### Co-immunoprecipitation and Western blot

Logarithmically growing cells were harvested and frozen at −80°C. Pellets were resuspended in the same volume of HB2 buffer (50 mM HEPES/KOH at pH 7.5, 140 mM NaCl, 15 mM EGTA, 15 mM MgCl_2_, 0.1% NP-40, 0.5 mM Na_3_VO_4_) containing protease inhibitors (1 mM dithiothreitol, 1 mM PMSF, 0.1% Protein inhibitor cocktail set III (Sigma), 0.1 ng/ml MG132 (Sigma), 10 U/ml TURBO DNase (Ambion)). Cells were broken using a Fast Prep machine (Thermo), briefly sonicated using the Biorupter and centrifuged to harvest the chromatin containing cell extract. Monoclonal anti-Flag M2 antibody (Sigma), anti-Myc 9E11 (Cell Signalling), anti-PK antibody (AbD Serotec/Bio-Rad) and anti-HA antibody (Covance) were used for pull down and detection of FLAG, Myc, PK and HA tagged proteins respectively. Anti-Cdc2 antibody (Santa Cruz) was used as a control. For protein complex immunoprecipitation, antibodies were conjugated with mouse IgG-coated Dynabeads (Life Technologies).

### Chromatin immunoprecipitation (ChIP)

Trt1 ChIP was performed as described previously (11). Cells were fixed in YES media containing fixing solution (1% formaldehyde, 10 mM sodium chloride, 0.1 mM EDTA, 0.05 mM EGTA and 5 mM Tris–HCl pH 8.0) for 15 min before harvesting cells. Whole cell extracts were sonicated and normalized. The PK epitope-tagged Trt1 complex was immunoprecipitated with the IgG-beads conjugated anti-PK antibody. For ChIP efficiency, co-precipitated DNA was quantified by quantitative PCR method using real-time PCR (SyberGreen and LightCycler 480, Roche) with a primer set ‘Telo top’ 5′-CGGCTGACGGGTGGGGCCCAATA-3′ and ‘Telo bot’ 5′-GTGTGGAATTGAGTATGGTGAA-3′ for the telomeres and a primer set ‘act1 F150’ 5′-GGATTCCTACGTTGGTGATGA-3′ and ‘act1 R277’ 5′- CGTTGTAGAAAGTGTGATGCC-3′ for the internal control *act1*. The water control gave a negative result. Efficiency of qPCR for the telomere and *act1* was on average 1.969 and 1.976 from three experiments, respectively. Absolute quantification was determined from mean CT value from triplicated real-time PCR using the standard curve. ChIP efficiency was calculated after normalising against input, and the efficiency of Trt1-PK binding to telomeres was expressed as the fold enrichment compared with the value obtained from cells harbouring untagged protein. Mean fold-enrichment, the standard deviation and the statistical significance were assessed from three individual experiments.

### Reverse-transcription quantitative PCR (RT-qPCR)

RT-qPCR for TERRA detection was modified from the described RT-PCR method (48). RNA was harvested from logarithmically growing cells (using Fungal RNA MiniPrep kit, Zymo Research), yielding approximately 50 μg total RNA per sample. Genomic DNA was removed by treatment with 10 U/ml RNase-free TURBO DNase (Ambion). 1 μg RNA was reverse-transcribed using 200 U SuperScript IV Reverse Transcriptase (ThermoFisher Scientific) in a 20 μl reaction using 50 μm random hexamer oligos, according to the manufacturers guidelines. RT products were diluted to 200 μl with DEPC treated water and RNA was removed with RNase A. Samples were stored at –80°C between use. RNA transcription was quantified using real-time PCR in a 20 μl reaction with 5 μl RT product, 2× SYBR Green PCR Master Mix (Roche) and 5 μM of each oligo, according to manufacturers guidelines. Reactions were run on a LightCycler 480 (Roche) under the following conditions: 95°C for 10 min followed by 40 cycles of 10 s at 95°C, 20 s at 60°C and 10 s at 72°C. Melting curve analysis was performed immediately after. Primer sets were ‘TERRA o1’ 5′-TAGGAAGTGCGGTAAGTTG-3′ and ‘TERRA o4’ (the same oligo as ‘Telo top’) for TERRA (48) and ‘snR101 F5’ 5′- TTCGCACATGGAATGGTTCA-3′ and ‘snR101 R145’ 5′- ACAGTGTAAGCTAGAACCAG-3′ for an 140 bp amplicon of the box H/ACA small nucleolar RNA snR101 as reference. Efficiency of qPCR for TERRA and snR101 from three experiments was on average 2.08 and 1.91 respectively. The expression level of TERRA was normalized to snR101 using the ΔCT method and expressed as ΔΔCT compared with wild type. Average was calculated from three biological replicas and unpaired t-test analyses were performed to determine significance.

### Yeast two-hybrid assay

The assay was conducted according to the Matchmaker gold yeast two hybrid system manual (Clontech). Budding yeast were grown in budding yeast synthetic media (SD: FORMEDIUM™). A series of truncated *ccq1* genes were cloned and fused to the gene encoding the GAL4 activation domain and the genes encoding the interaction proteins were cloned and fused to the gene encoding the GAL4 DNA binding domain. Expression of the GAL domain bound proteins was confirmed by western blot using anti-myc and anti-HA antibodies. Before spotting on the plate, cells were cultured in SD-LW media to logarithmic growth and equal volumes of cells were spotted. Plates were incubated at 28°C for 2–3 days before photography. Strength of the protein interaction was assessed using plates lacking both adenine and histidine or lacking histidine with 1 mM 3-amino-1,2,4-triazole (3-AT) as described in the corresponding figure legends.

### Gene silencing assay

To monitor transcription at the centromeric and telomeric regions, we utilised cells carrying the auxotrophic markers, *ade6*^+^ and *ura4*^+^, at an outer repeat of chromosome 1 and a telomere-adjacent region of the left arm of chromosome 2, respectively (51). As cells carry the *ade6-M210* mutation at the endogenous gene locus, cells become red when centromeric transcription is repressed. Similarly, expression of the telomeric *ura4^+^* gene leads to a growth defect in the presence of 5-FOA. Before spotting on the plate, cells were cultured in EMM media to logarithmic growth and equal volumes of cells were sequentially diluted and spotted on EMM plates with full supplements (input control), lacking adenine and supplied with 5-FOA (with low uracil). Plates were incubated at 32°C for 2–3 days before photography.

### Microscopy

Freshly growing cells on YES plates were prepared on glass slides with yeast minimum media. Imaging was carried out with a DeltaVision Elite (Applied Precision) comprising an Olympus IX71 inverted fluorescent microscope, and Olympus UPlanSApo 100×, NA1.40, oil immersion objective and a CoolSNAP HQ2 camera. mCherry, YFP and Cerulean signals from cells were captured with 0.2 s (32% filter), 0.4 s (32% filter) and 0.3 s (32% filter) exposures per plane at a 0.3 μm step size over 11 focal planes (z-sections), respectively. These images were deconvolved and analysed using SoftWoRks (Applied Precision). Cell images at the original focal point were also captured using DIC (differential interference contrast) as a reference.

### Cell cycle arrest and FACS analysis

To arrest cells at S-, G2- and G1-phases, hydroxylurea (HU) and *cdc25–22* and *cdc10-V50* strains were used, respectively. Briefly, wild type cells and *ts* mutants (*cdc25–22, cdc10-V50*) were cultured in 100 ml YES media at 25°C to log phase (up to 1.0 OD^600^). Then the cultures were diluted to 0.5 OD by adding YES preheated to 35°C. For S-phase arrest, HU was added to the wild type cell culture with 12 mM HU final concentration. All the cultures were incubated at 35°C for 3 h. 5 ml of each culture were fixed in ethanol and stained with propidium iodide and subjected to DNA content analysis by FACS using the BD LSRFORTESSA X-20 (BD FACS Diva software).

## RESULTS

### Ccq1 has a nuclear localisation signal

**Figure 1.**
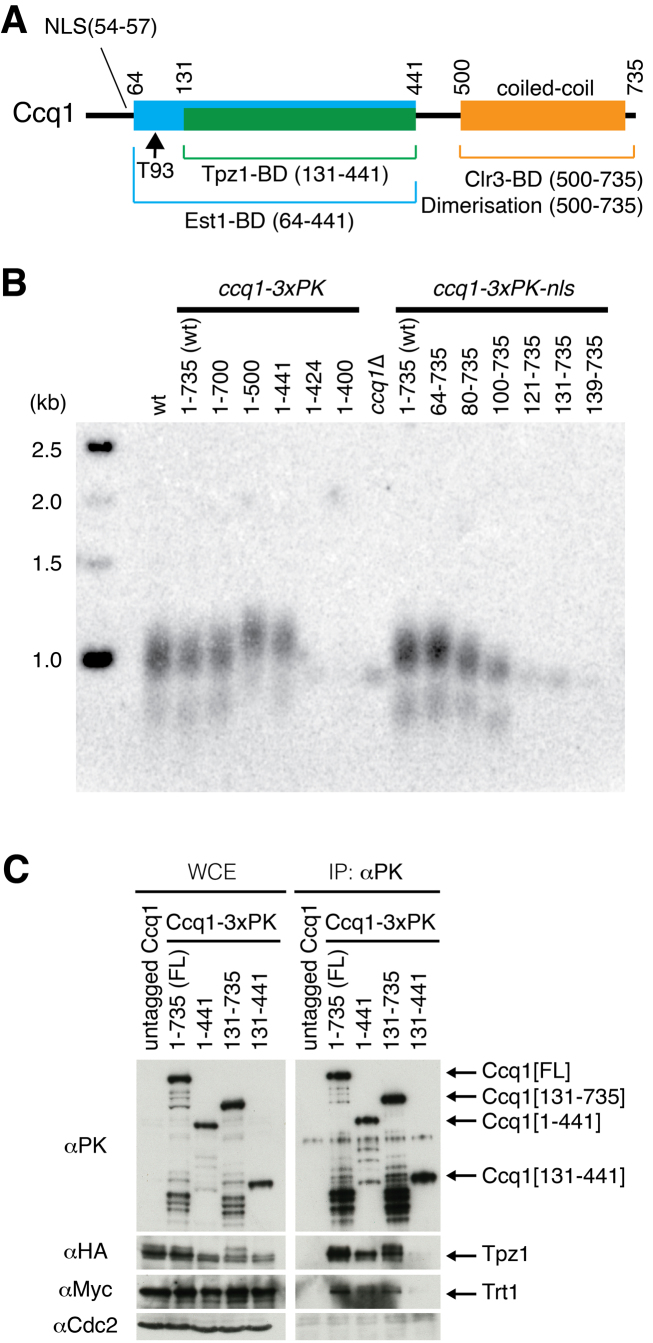
Ccq1 C-terminus negatively controls telomere lengthening. (**A**) Schematic diagram of Ccq1 domains. The protein interaction regions were primarily assessed by the yeast two hybrid system. The core domain of Ccq1 stems from the region 131–441 residues that interacts with Tpz1 and is essential for any function of Ccq1. The Est1 binding domain contains residues including Threonine 93 and the Tpz1-binding domain and therefore begins from 64th amino acids and includes the Tpz1 binding site. The Ccq1 dimerization and Clr3 binding domain is located on the C-terminus (amino acids 500–735), which includes the coiled-coil motifs. (**B**). Telomere Southern blot shows that truncation of the coiled-coil motifs in Ccq1 leads to slight elongation of telomeres. The *ccq1(1–500)* and *(1–441)* truncation mutants exhibited slightly longer telomeres, but further C-terminal truncation or truncation of the Est1 binding domain resulted in maintenance of short telomeres, equivalent to *ccq1*Δ. *Eco*RI digested telomere fragments are an average length of 1 kb in wild type cells. (**C**) Co-immunoprecipitation of the PK epitope-tagged Ccq1 truncations revealed association with Tpz1 and Trt1. Although the Ccq1(131–441) truncation interacted with Tpz1 in the yeast two-hybrid system (Supplementary Figure S2A), neither this interaction nor telomerase association could be detected by co-immunoprecipitation. Slower migrating bands of Tpz1 are presumably caused by phospho-modifications, which are lost in *ccq1(1–441)* and *ccq1(131–441)* backgrounds.

To begin analyzing the role of Ccq1 in telomere length homeostasis, we first dissected the protein. Ccq1 is predominantly alpha helical, with a predicted coiled-coil motif at the C-terminus (amino acids 500–700, Figure 1A) (52). The N-terminus of Ccq1 carries a cluster of basic residues, a predicted nuclear localisation signal (NLS). Whereas wild type Ccq1 localises only to the nucleus, point mutations within this region (K54A and R55A) led to diffusion of Ccq1 throughout the cytoplasm, confirming this sequence as an NLS (Supplementary Figure S1A). Nevertheless, Ccq1 foci were retained at telomeres and telomere length was not impaired in the *ccq1-*Δ*nls* mutants (Supplementary Figure S1). We therefore concluded that the NLS within Ccq1 is not required for its localisation to the telomeres. Rather, Ccq1 may be transported and recruited to telomeres *via* other shelterin complex components, such as Tpz1.

### Truncation of Ccq1 C-terminus leads to elongation of telomeres

Tpz1 binds to the core region of Ccq1 (amino acids 131–441, Figure 1A). Est1 binds to two regions of Ccq1; the N-terminal region including the phosphorylated threonine 93 residue (13) and the region including residues 157–175, which lies within the Tpz1-binding domain (30). Accordingly, our yeast two-hybrid analysis confirmed previous reports that Est1 binds weakly to the Tpz1 binding domain (amino acids 131–441, Supplementary Figure S2A). Further truncation of the C-terminus diminished the interaction with Est1, suggesting that the entire Tpz1-binding domain is required for Est1 interaction. The strength of the interaction was not increased in Ccq1(63–441) but was in the full length Ccq1, which includes the C-terminal coiled-coil motifs. Hence, the C-terminus of Ccq1 appears to strengthen the interaction with Est1 in the yeast two-hybrid assay.

To gain insight into how Ccq1 controls telomere length homeostasis, we generated a series of *ccq1* mutant strains that express truncated Ccq1 from the endogenous locus (Figure 1B and Supplementary Figure S2B). In the case of N-terminal truncations, an ectopic NLS was fused to ensure nuclear localisation was not impaired. This analysis revealed no change in telomere length upon the loss of amino acids 1–63, suggesting that the region containing the NLS is not required for telomere maintenance (Figure 1B: lane [64–735]). However, loss of further N-terminal residues led to telomere shortening (lanes [80–735] and [100–735]). Interestingly, truncations of the C-terminus to eliminate the coiled-coil motifs (lanes [1–441] and [1–500]) led to slight elongation of telomeres, implying that the C-terminus of Ccq1 is involved in negatively regulating telomere extension. This was unexpected as Ccq1 was thought to act solely as a positive regulator of telomerase and contradicted current knowledge (41). The series of Ccq1 truncations were relatively well expressed compared to full length Ccq1, except for the 1–424 and 1–400 fragments, which had significantly reduced expression (Supplementary Figure S2B). The telomere elongation phenotype observed in the *ccq1(1–441)* truncation mutants was not associated with the fused epitope tags, as both PK and Flag tagging caused a similar effect (Supplementary Figure S2C). Although dysfunction of Ccq1 leads to homologous recombination-mediated telomere maintenance (26), the *ccq1(1–441)* truncation mutants maintained telomere integrity in the absence of Rad51 (Supplementary Figure S4). Instead, loss of telomeres was observed in the absence of TER1, indicating telomerase dependent elongation. Thus, the N-terminus and C-terminus of Ccq1 are involved in positive and negative regulation of telomerase activity, respectively.

To determine if the Ccq1 truncations were able to associate with Tpz1 and the telomerase catalytic subunit Trt1, co-immunoprecipitation was performed (Figure 1C). Accordingly, both the C-terminally truncated Ccq1(1–441) and N-terminally truncated Ccq1(131–735) associated with Tpz1, as these fragments encompass the Tpz1-binding domain (Figure 1C). Further truncation of the N-terminus leads to loss of the interaction with Tpz1 (Supplementary Figure S2D). However, contrary to the yeast two-hybrid result, the identified Tpz1-binding region alone (amino acids 131–441) did not co-immunoprecipitate with Tpz1 in the cell extract (Figure 1C), suggesting that a larger region of Ccq1 is required for a stable association with Tpz1. Interestingly, the telomerase catalytic subunit Trt1 could be co-purified with the Ccq1(131–735) truncation, which lacks a part of the Est1 binding domain (Figure 1C). Previous work from our lab indicated that Trt1 forms an alternative stable association with Tpz1 and Ccq1 (11). As such, we speculate that Ccq1 can associate with Trt1 not only when it is in complex through the interaction with Est1 and TER1. Collectively, the data indicate that the N-terminus of Ccq1 (amino acids 64–441) is crucial for telomere maintenance and is essential for association with Est1. Conversely, the C-terminus of Ccq1 (amino acids 131–735) is able to associate with Trt1 but is not functional for telomere elongation.

### Ccq1 and Est1 fusion bypasses the telomerase recruitment step

**Figure 2.**
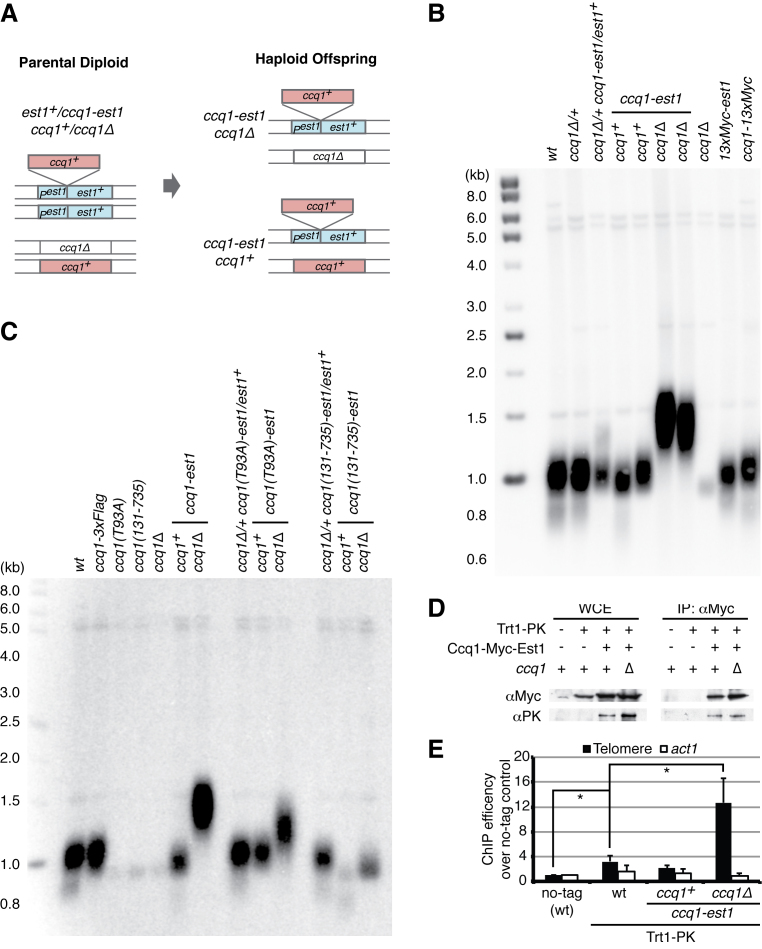
The Ccq1-Est1 fusion bypasses the recruitment step. (**A**) Schematic diagram of the fusion system. The *ccq1* gene sequence with thirteen tandem *myc* sequences was inserted after the start codon of one *est1* allele in a diploid strain heterozygous for *ccq1*Δ. Expression of the intact chimera gene product (Ccq1–13xMyc-Est1) through the endogenous *est1* promoter was confirmed using anti-Myc antibody (Supplementary Figure S3). After sporulation, haploids were obtained which expressed the chimera protein from the *est1* locus in a *ccq1*Δ or *ccq1^+^* background. (**B**) Telomere Southern blot shows that expression of the Ccq1-Est1 fusion protein leads to longer telomere maintenance in the absence of endogenous Ccq1. 13xMyc epitope tagging to N-terminus of Est1 and the C-terminus of Ccq1 did not impair function in telomere length homeostasis. (**C**) Telomere Southern blot shows that the Ccq1-Est1 chimera bypasses need for phosphorylation at Ccq1 Thr93 for telomere maintenance but truncation of Ccq1 N-terminus (131–735) leads to maintenance of short telomeres. In the presence of the Ccq1(131–735)-Est1 chimera, telomere lengthening was severely impaired in *ccq1*^+^ background. Nevertheless, elongation of telomeres was observed in the *ccq1*Δ background, suggesting that negative regulation by endogenous Ccq1 remains functional. (**D**) Co-immunoprecipitation with the Ccq1–13xMyc-Est1 chimera shows stable association with Trt1. (**E**) Chromatin immunoprecipitaion (ChIP) with the PK epitope-tagged Trt1 shows efficient recruitment of telomerase to telomeres in the presence of the Ccq1-Est1 chimera and the absence of endogenous Ccq1. DNA fragments containing telomere-adjacent sequence and the *act1* gene (as internal control) were detected by quantitative PCR. ChIP efficiencies of telomeric DNA and *act1* were calculated against input (whole cell extract), and the fold enrichment over no-tagged negative control is expressed. Mean average of three biological replicas is shown. Significant differences over Trt1-PK (wild type) are indicated as asterisks (two-tailed *t*-test: *P < 0.05).

Previous studies suggested that Ccq1 is required for both telomerase recruitment and the following activation step (11,25,26). To investigate events after telomerase recruitment, the *ccq1^+^* coding sequence and a linker, encoding a 13-tandem Myc-epitope tag, were inserted at the beginning of the endogenous *est1* gene to express a Ccq1–13xMyc-Est1 chimera protein in a diploid strain (Figure 2A: *ccq1-est1* indicates the fusion gene encoding Ccq1–13xMyc-Est1). Both *est1* and *ccq1* genes are essential for telomerase activity and telomere length homeostasis, and deletion of either gene leads to loss of telomeres (25,26,53). The diploid cells and their haploid offspring carrying the fused *ccq1-est1* allele maintained telomeres at lengths comparable to wild-type cells (Figure 2B: lanes *ccq1*^+^ in *ccq1-est1*). Further deletion of the endogenous *ccq1* gene did not cause telomere loss (Figure 2B: lanes *ccq1*Δ in *ccq1-est1*). Rather, intriguingly *ccq1* deletion mutants expressing the Ccq1-Est1 chimera harbour elongated telomeres. Accordingly, telomeres in cells expressing the Ccq1-Est1 chimera were maintained by telomerase but not *via* homologous recombination (Supplementary Figure S4). We therefore inferred that the Ccq1-Est1 chimera was able to retain each protein's function for telomere elongation.

The telomerase recruitment step relies on phosphorylation of Ccq1, especially at threonine 93, which provides affinity with Est1 (13,18,29). Hence, the *T93A* mutation of *ccq1*^+^ leads to an ‘ever shorter telomere’ phenotype, like that of *est1*Δ. Importantly, fusion of Est1 with Ccq1-T93A rescued this telomere-shortening defect (Figure 2C), further confirming that the Ccq1-Est1 chimera bypasses the Ccq1 phosphorylation-dependent Est1 recruitment step. However, N-terminal truncation of Ccq1 (amino acids 131–735), that lost part of the Est1 binding domain, was found to cause impaired telomere lengthening, even when fused to Est1 (Figure 2C). Further reduction in telomere length in the presence of endogenous Ccq1 suggested that overall telomerase activity was impaired. The differing results between the *ccq1-T93A* mutation and the *ccq1(131–735)* truncation suggest that the Ccq1 N-terminus has a crucial role in telomerase elongation, beyond telomerase recruitment *via* Est1.

### The Ccq1-Est1 chimera recruits telomerase to telomeres

The efficiency of telomerase association with the Ccq1-Est1 chimera was assessed. Co-immunoprecipitation indicated that the Ccq1-Est1 chimera associates with Trt1 (Figure 2D). In addition, telomere chromatin immunoprecipitation (ChIP) with Trt1 indicated that the Ccq1-Est1 chimera recruits telomerase to telomeres at a similar level to wild type cells in the presence of endogenous Ccq1 (Figure 2E). However, in the absence of endogenous Ccq1, telomerase recruitment was significantly more efficient than in wild type cells. Thus, our immunoprecipitation assays confirmed that the Ccq1-Est1 chimera associates well with Trt1 and maintains telomeres, and the ChIP assay suggests that endogenous Ccq1, whilst not involved in telomerase recruitment, plays a role in negative telomerase regulation.

After telomerase is recruited to the telomeres, telomerase activity relies on the OB-fold domain of Tpz1. The *tpz1* mutation *K75A* leads to telomere shortening without impairing the recruitment step (11). Introduction of the *K75A* mutation in *tpz1* in the strain expressing the Ccq1-Est1 chimera caused shortening of telomeres, like the *tpz1-K75A* single mutant (Supplementary Figure S5). This suggests that the role of Tpz1 in recruiting and activating telomerase is downstream of the Ccq1 and Est1 interaction.

### Ccq1 negatively regulates telomere lengthening *via* its Tpz1-binding and C-terminal coiled-coil domains

**Figure 3.**
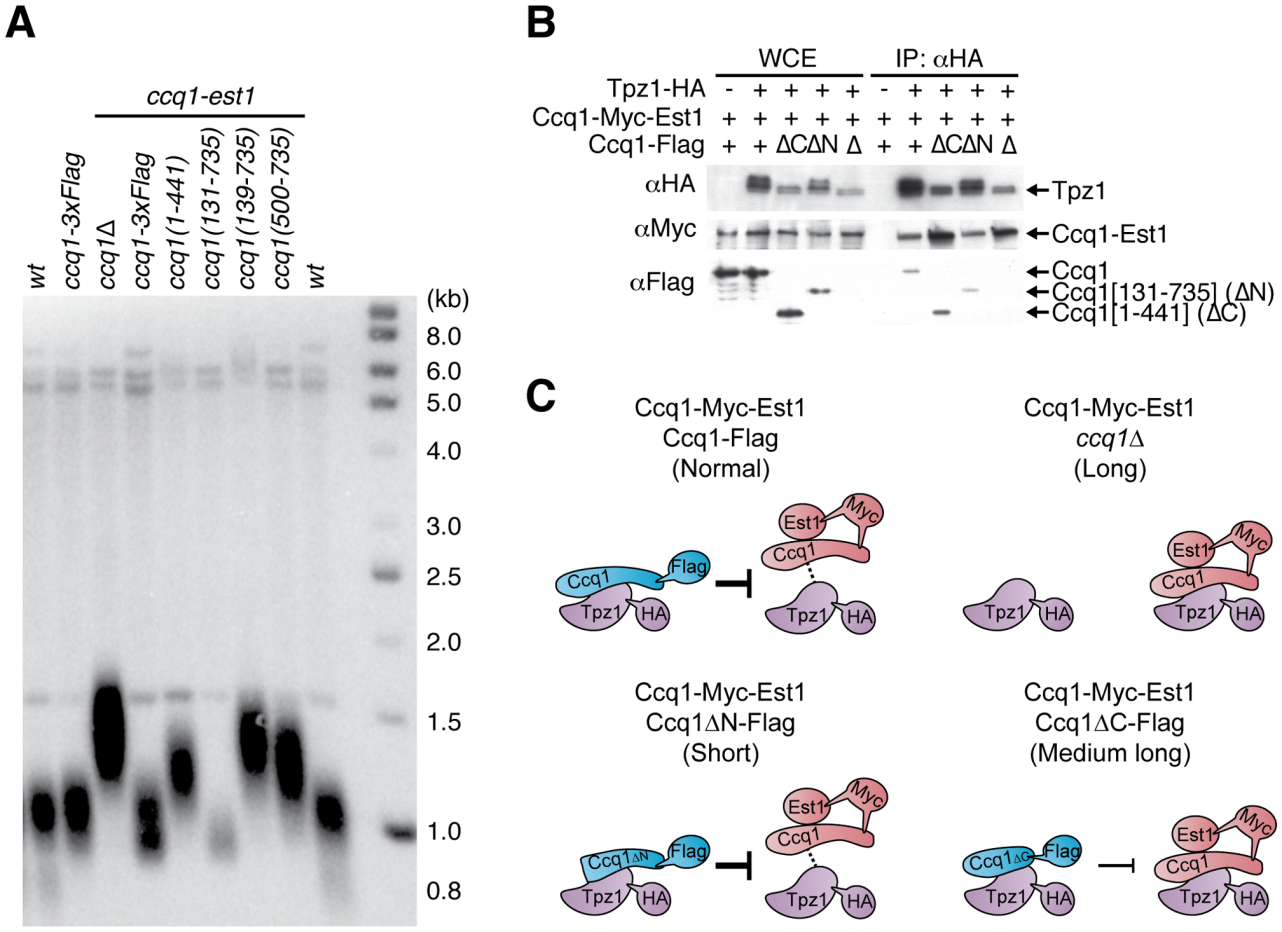
Telomerase free Ccq1 negatively regulates telomere length *via* its Tpz1-binding and C-terminal domains. (**A**) Telomere Southern blot shows that Ccq1 C-terminus truncation (1–441) leads to telomere elongation, like *ccq1*Δ, in the presence of the Ccq1-Est1 chimera. Conversely, the N-terminus truncation (131–735) leads to telomere shortening but further truncation over the Tpz1 binding domain (139–735 and 500–735) leads to loss of negative regulation. (**B**) Both the Ccq1-Est1 chimera and the truncated Ccq1 (ΔC: amino acids 1–441; ΔN: amino acids 131–735; Δ: null) were co-immunoprecipitated with the HA epitope-tagged Tpz1. Stable association between the Ccq1-Est1 chimera and Tpz1 was observed in the absence of endogenous Ccq1 (Δ) and in the presence of the C-terminus truncated Ccq1 (ΔC). Slower migrating bands of Tpz1 are presumably caused by phospho-modifications, which are lost in *ccq1*Δ and *ccq1(1–441)* backgrounds that cause a telomere elongation phenotype. (**C**) Schematic diagram describing Ccq1 association with Tpz1 in cells expressing both endogenous (full-length or truncated) Ccq1, and the Ccq1-Est1 chimera. The telomere status is indicated in the brackets. The presence of free Ccq1 weakens the association between Tpz1 and the Ccq1-Est1 chimera (represented as dashed lines). This inhibitory effect is reduced by Ccq1 with a C-terminal deletion (represented as thinner block dash line).

Elimination of endogenous Ccq1 in strains expressing the Ccq1-Est1 chimera led to efficient telomerase recruitment and elongation of telomeres, indicating that endogenous Ccq1 is able to suppress the function of the Ccq1-Est1 chimera. Although Ccq1 is an abundant protein compared to Tpz1 (41), the negative regulation by endogenous Ccq1 was not simply caused by competitive occupation of Tpz1 against the Ccq1-Est1 chimera. Truncation analysis of endogenous Ccq1 showed that this elongation effect was also seen in strains with a C-terminal truncation of endogenous Ccq1 (amino acids 1–441) (Figure 3A: lane *ccq1(1–441)* in *ccq1-est1*), suggesting that a negative regulatory effect requires the Ccq1 C-terminus. Conversely, the N-terminus region is not required for the negative regulation (lane *ccq1(131–735)* in *ccq1-est1*). However, further truncation over the Tpz1-binding domain did cause telomere lengthening and loss of the negative regulation effect (lanes ccq1*(139–735) and (500–735)* in *ccq1-est1*).

Co-immunoprecipiation assays indicated that Tpz1 was able to interact well with both the N- and C-terminal truncations of endogenous Ccq1 (Figure 1C). In the presence of the Ccq1-Est1 chimera, the interaction between Tpz1 and truncated Ccq1 was retained (Figure 3B). However, Tpz1 interacted more efficiently with the Ccq1-Est1 chimera in the absence of endogenous Ccq1 or in the presence of the C-terminally truncated Ccq1 (amino acids 1–441) (Figure 3C). Taken together, these data indicate that the inhibitory effect by endogenous Ccq1 requires the Tpz1-binding domain and is more effective in the presence of the C-terminal region.

Telomerase recruitment is restricted by interaction of the Stn1–Ten1 complex with SUMOylated Tpz1 (33,34). Hence, deletion of the SUMO peptide encoding gene, *pmt3*, or *tpz1-K242R* mutation, results in elongation of the telomeres. As the Tpz1-binding domain of Ccq1 is crucial for the negative regulation observed in our study, the involvement of this SUMO-mediated telomerase termination pathway was assessed. We observed additive elongation of telomeres in *tpz1-K242R ccq1(1–441)* double mutants, indicating two independent restriction mechanisms for telomere elongation. Additive telomere elongation was also observed in *tpz1-K242R ccq1Δ* cells expressing the Ccq1-Est1 chimera, and in the *pmt3Δ* mutant background (Supplementary Figure S6). Thus, the Tpz1-SUMO-Stn1–Ten1-mediated function does not seem to be required for Ccq1-dependent telomerase suppression.

### Ccq1 forms a dimer/multimer *via* its coiled-coil domain

**Figure 4.**
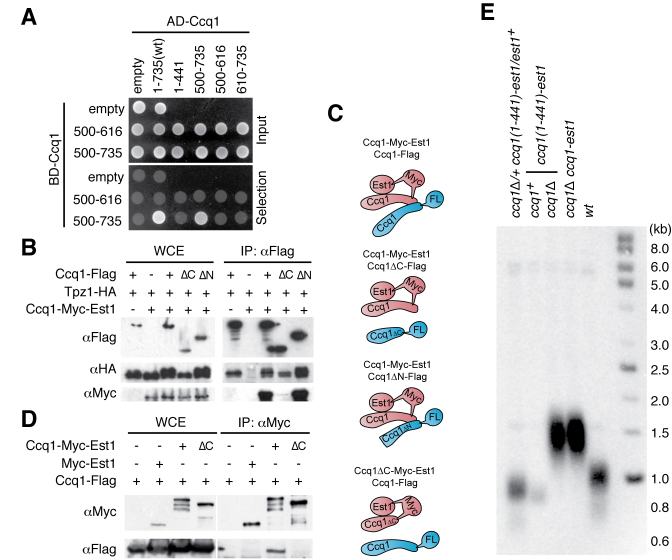
The coiled-coil motifs region of Ccq1 forms a homodimer/multimer. (**A**) The yeast two-hybrid assay shows that the C-terminal coiled-coil motifs (500–735) forms a dimer/multimer. The indicated Ccq1 truncation proteins were fused to the GAL4 activation domain (AD) and the C-terminus fragments of Ccq1 were fused to the GAL4 DNA binding domain (BD). Selection plate lacks adenine and histidine. (**B**) The Ccq1-Est1 chimera and Tpz1 were co-immunoprecipitated with the FLAG epitope-tagged truncated Ccq1 (ΔC: 1–441aa; ΔN: 131–735aa). While Tpz1 interacted with both Ccq1 truncations, the C-terminus truncation failed to interact with the Ccq1-Est1 chimera. (**C**) Summary representation of co-immunoprecipitation study in B and D. Ccq1-Flag and Ccq1-Myc-Est1 interacts *via* their Ccq1 C-terminal domain. (**D**) Co-immunoprecipitation of the Myc epitope-tagged Est1 and the Ccq1-Est1 chimera (ΔC: Est1 is fused to Ccq1(1–441), the C-terminus truncation) showed that the Ccq1-Est1 chimera associated with Ccq1 *via* its C-terminus. (**E**) Telomere Southern blot shows that truncation of the Ccq1 C-terminus region within the Ccq1-Est1 chimera did not impair telomere lengthening. The elongation of telomeres by the Ccq1(1–441)-fused-Est1 chimera was similar to that by full length Ccq1-Est1 chimera in the absence of endogenous Ccq1.

The C-terminus of Ccq1 contains coiled-coil motifs, which can form dimeric/trimeric complexes (54). Accordingly, our yeast two-hybrid assay revealed that the C-terminus of Ccq1 (amino acids 500–735) formed a homodimer or multimer (Figure 4A). Co-immunoprecipitation experiments showed that full length Ccq1 and the N-terminally truncated Ccq1 (amino acids 131–735, ΔN) associated with both the Ccq1-Est1 chimera and Tpz1, but Ccq1(1–441), which lacks the C-terminus coiled-coil motifs (ΔC), failed to associate with the Ccq1-Est1 chimera (Figure 4B, C). This result suggests that endogenous Ccq1 associates with the Ccq1-Est1 chimera through its C-terminus but not through Tpz1.

To address if Ccq1 dimerization is associated with negative regulation of telomere lengthening, the C-terminal region of Ccq1 was removed from the Ccq1-Est1 chimera. As expected, the Ccq1(1–441)-Est1 chimera did not interact with endogenous Ccq1 (Figure 4C and D). However, telomeres in cells expressing the Ccq1(1–441)-Est1 chimera were elongated to the same extent as for the full length Ccq1 fusion with Est1 (Figure 4E). This suggested that the coiled-coil motifs are not required for telomere lengthening and the dimerization/multimerization of Ccq1 is dispensable from its negative regulation.

### Clr3 interacts with Ccq1 at the coiled-coil region and counteract telomere elongation

As Ccq1 binds to Tpz1 and is involved in regulation of shelterin formation (25,27,55), we asked whether the C-terminus of Ccq1 participates in controlling whether telomeres are in an extendible or non-extendible state. Deletion of genes encoding the shelterin components Taz1, Rap1 and Poz1, as well as the Taz1 binding protein Rif1 (which is not a part of shelterin), leads to deregulated telomerase recruitment and elongation of telomeres (4,25,56). We deleted the *rif1* gene in cells expressing the Ccq1-Est1 chimera, and this caused an additive telomere elongation phenotype (Supplementary Figure S7), indicating distinct mechanisms for telomere length control. However, disruption of shelterin formation by eliminating the shelterin protein Rap1 in cells expressing the Ccq1-Est1 chimera caused a long telomere phenotype similar to the single *rap1Δ* mutants (Supplementary Figure S7). This suggests that shelterin formation is required for Ccq1-mediated telomere length control.

**Figure 5.**
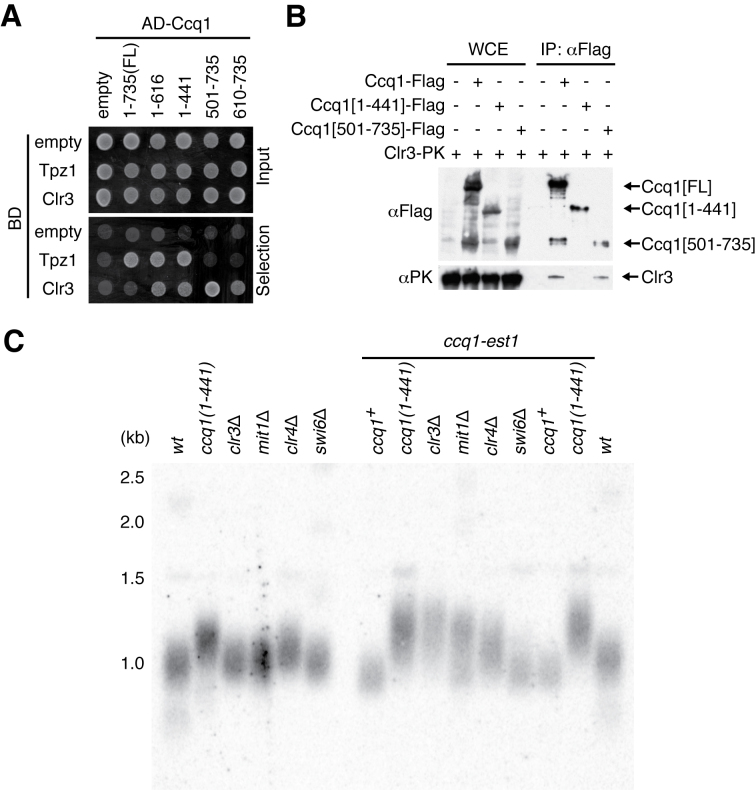
Ccq1 dependent negative regulation of telomere lengthening *via* Clr3-SHREC. (**A**) The yeast two-hybrid assay shows that Clr3 interacts strongly with Ccq1(500–735) and weakly with Ccq1(1–441). The indicated Ccq1 truncation proteins were fused to the GAL4 activation domain (AD) and Tpz1 and Clr3 were fused to the GAL4 DNA binding domain (BD). Selection plate lacks adenine and histidine. (**B**) Co-immunoprecipitation with the truncated Ccq1 showed that endogenously expressed Ccq1 associates with Clr3 *via* its C-terminus (amino acids 500–735). (**C**) Telomere Southern blot shows mild telomere elongation in *clr4*Δ, similar to *ccq1(1–441)*. Deletion of *clr3* and *mit1* leads to elongation of telomeres only in the presence of the Ccq1-Est1 chimera.

Whilst shelterin formation is required for telomeric gene silencing and heterochromatin assembly (28,45,57), Rif1 is dispensable for these processes (56). Ccq1 is known to bind Clr3 to recruit SHREC to telomeres in a shelterin-dependent manner (37). Clr3 represses local transcription and maintains heterochromatin formation (39). Hence, we reasoned that Ccq1-dependent heterochromatin formation may suppress telomere lengthening. Our yeast two-hybrid analysis determined that two regions of Ccq1 interact with Clr3: Ccq1(1–441) and the coiled-coil motifs (Figure 5A). Whereas full length Ccq1 did not appear to interact with Clr3, the coiled-coil motifs containing region of Ccq1 (amino acids 500–735), which forms a dimer/multimer, strongly interacts with Clr3 in our assay. Co-immunoprecipitation assays indicate that association of Ccq1 with Clr3 is dependent on the C-terminus (Figure 5B). Thus, we conclude that Ccq1 recruits SHREC through its coiled-coil motifs.

To assess whether the negative regulation of telomere lengthening by Ccq1 is dependent on its interaction with Clr3 and/or heterochromatin formation, *clr3, mit1, clr4* and *swi6* were deleted from cells expressing the Ccq1-Est1 chimera. *mit1* encodes a chromatin remodelling factor within SHREC (37). Whereas deletion of *clr3* or *mit1* did not change telomere length in cells of a wild type background, telomeres were elongated when *clr3* or *mit1* were deleted in cells expressing the Ccq1-Est1 chimera (Figure 5C). This extension of telomeres was similar to that observed for the *ccq1(1–441)* truncation mutant. Further deletion of the *clr3*^+^ gene in *ccq1*Δ cells expressing the Ccq1-Est1 chimera slightly increased telomere length (Supplementary Figure S8). As telomere elongation was not fully additive, we speculate that Clr3-SHREC functions partly *via* Ccq1 to suppress lengthening of telomeres after Est1 recruitment. The Ccq1-independent activity of Clr3 may originate from the residual presence of heterochromatin proteins on the telomere-adjacent subtelomeric region. Interestingly, *clr4*Δ cells but not *swi6*Δ cells contained slightly longer telomeres, comparable to those of *ccq1(1–441)* cells (Figure 5C). Mild telomere elongation was observed in *clr4*Δ strains in the presence of the Ccq1-Est1 chimera (Figure 5C, Supplementary Figure S8). Furthermore, telomere length in *clr4*Δ *ccq1(1–441)* double mutants indicates that the *clr4* deletion and the *ccq1* C-terminal truncation mutant function epistatically (Supplementary Figure S8). The differing telomere phenotypes observed in *clr3*Δ and *clr4*Δ cells suggest that SHREC and the Clr4 complex function distinctly in telomere maintenance. Thus, we conclude that Ccq1 interacts with Clr3 and Clr4 to restrict telomere extension. This function is separate from the role of Ccq1 in telomerase recruitment.

### Ccq1 C-terminus represses telomere-adjacent transcription

**Figure 6.**
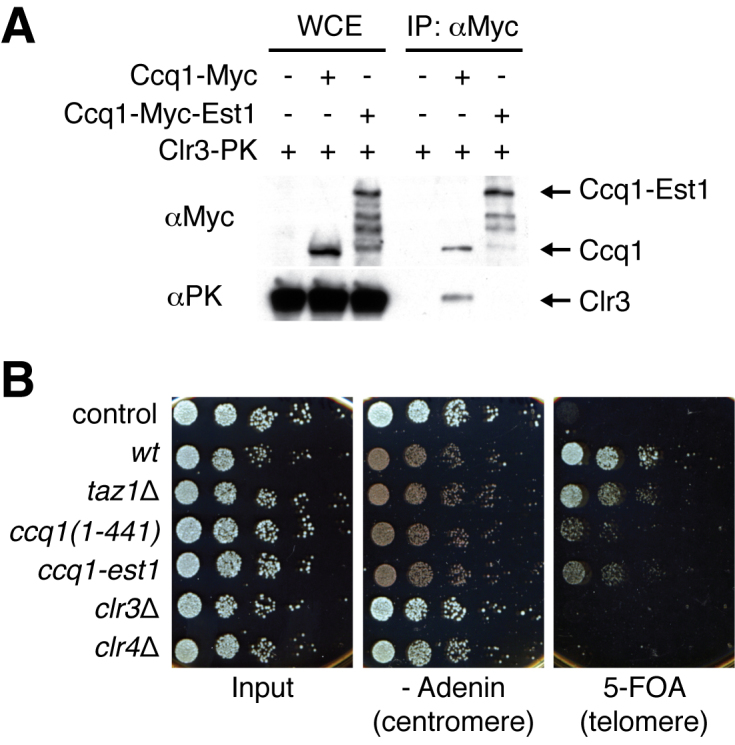
Clr3 associates with Est1-unbound Ccq1 and represses telomere-adjacent transcription. (**A**) Clr3 was co-immunoprecipitated with the Myc-epitope tagged Ccq1 but not with the Ccq1-Est1 chimera (in *ccq1*Δ background). Presence of endogenous Est1-free Ccq1 further reduced the interaction between Clr3 and the Ccq1-Est1 chimera (Supplementary Figure S10). (**B**) The heterochromatic gene-silencing assay. Serial dilution spot assay to measure transcription efficiencies of genes inserted at the centromere and telomere. The wild type *ade6^+^* gene cassette was inserted at a centromeric outer repeat region of chromosome 1, and the wild type *ura4^+^* gene cassette was inserted at the telomere on the left arm of chromosome 2. Centromeric repression of the *ade6^+^* gene leads to a growth defect and red colouring, and telomeric repression of the *ura4^+^* gene leads to survival of cells under 5-FOA treatment. Control cells are prototrophic, expressing both Ade6 and Ura4 (lane 1). The *taz1* deletion or *ccq1(1–441)* truncation mutation leads to activation of telomeric transcription, whereas deletion of *clr3* or *clr4* leads to gene silencing defects at both telomeres and centromeres.

Our Est1-fusion and Ccq1 truncation analyses reveal that Ccq1 is likely to form distinct complexes for positive and negative regulation of telomere lengthening. Hence, it was anticipated that Clr3 would associate with only the negatively regulating Ccq1 complex. Co-immunoprecipitation experiments showed that Ccq1 and Clr3 appear to form a constitutive complex throughout cell cycle (Supplementary Figure S9). However, whereas Clr3 associated with endogenous Ccq1, it could not be efficiently immunoprecipitated with the Ccq1-Est1 chimera (Figure 6A, Supplementary Figure S10). Thus, the interactions of Ccq1 with Est1 and Clr3 are mutually exclusive and we propose that they function to positively and negatively regulate telomerase activity respectively.

To measure the efficiency of heterochromatic gene silencing by the Ccq1-Est1 chimera and the Ccq1 C-terminus truncation, we utilised a strain carrying the *ura4^+^* auxotrophic marker next to telomeric repeats on chromosome 2 and the *ade6*^+^ marker at the centromeric region on chromosome 1 (Figure 6B) (51). These cells change colour from red to white when centromeric gene silencing is compromised, and 5-fluorootic acid (5-FOA) becomes toxic when telomeric gene silencing is compromised. Deletion of *clr3* and *clr4* leads to disruption of both telomeric and centromeric gene silencing, and deletion of *taz1* leads to disruption of only telomeric gene silencing (Figure 6B). A recent study showed that C-terminal truncation of Ccq1 recaptures the telomeric gene-silencing defect shown in *ccq1*Δ cells (41). We confirmed that our *ccq1(1–441)* truncation activated the telomere-adjacent gene *ura4*^+^, resulting in cellular sensitivity to 5-FOA. This suggests that the C-terminus of Ccq1 is required for repression of telomere-associated transcription, most likely *via* its association with Clr3 (and Clr4). Similarly, cells expressing the Ccq1-Est1 chimera were sensitive to 5-FOA (Figure 6B), supporting the idea that Ccq1 does not function in transcriptional repression when it is bound to Est1. Quantitative reverse-transcription PCR (RT-qPCR) showed a mild increase in endogenous transcription of TERRA in *ccq1(1–441)* and *clr3*Δ strains (Supplementary Figure S11). Collectively, we conclude that SHREC is recruited to the telomeric DNA *via* telomerase-unbound Ccq1 to reinforce local transcriptional repression at telomeres.

## DISCUSSION

The dual roles of the shelterin components Tpz1 and Ccq1 in both recruitment/retention and activation of telomerase makes the investigation of these mechanisms challenging. In this study, our Est1 fusion system enabled us to model events after the Ccq1-Est1 interaction step in telomerase recruitment. The data reveal that, together with SHREC and the Clr4 complex, Ccq1 participates in negative regulation of telomerase activity. The distinction between the telomerase recruitment and negative regulation functions of Ccq1 appears to be directed by its binding partners. Whilst Est1 binds to the N-terminus of Ccq1 to positively regulate telomerase, Clr3 binds to the C-terminus of Ccq1 most likely to negatively regulate telomerase. Although the binding regions of Est1 and Clr3 are distinct, these proteins appear to bind mutually exclusively to Ccq1. The negative regulation of telomere lengthening by Clr3 was observed only in the presence of the telomerase-associated Ccq1, suggesting that the Ccq1-Clr3 complex acts downstream of telomerase recruitment. Genetic interaction between *ccq1(1–441)* and *clr4*Δ suggests that Clr4 also functions in negative regulation of telomerase *via* the Ccq1 C-terminus. Thus, Ccq1 controls telomerase action *via* distinct complex formation.

As telomerase-dependent telomere elongation is controlled at multiple levels, by the recruitment process, the activation step and the rate of processivity, a layer of negative regulation is also to be expected. Shelterin controls the telomerase recruitment step, which is initiated by Ccq1 phosphorylation (4,27,58). Ccq1-dependent negative regulation of telomerase seems also to be dependent on shelterin. Our ChIP and co-immunoprecipitation data suggest that the association of Ccq1-telomerase with Tpz1 can be inhibited by the C-terminus of Ccq1, which binds to Clr3-SHREC, thus terminating telomerase activity by releasing Ccq1-bound telomerase. Dissociation of Ccq1 from Tpz1 is also supported by a previous report, in which regulation of the interaction between Tpz1 and Ccq1 was found to be involved in telomerase recruitment/retention (59). Hence, telomerase can be released from the telomere by dissociation of Est1 from Ccq1 (*via* de-phosphorylation of Ccq1) and by dissociation of telomerase-bound Ccq1 from Tpz1. Overall, we propose that following telomere extension, the Ccq1-SHREC complex releases the telomerase-bound Ccq1 from Tpz1 to terminate retention of telomerase (Supplementary Figure S12).

Mechanistically, how Clr3 and SHREC counteract telomere lengthening remains to be addressed. We suspect the chromatin-remodelling activity of SHREC is likely to be involved, as the Clr3-Ccq1 complex is an orthologue of the budding yeast HDA1–2-3 HDAC complex and the SHREC component Mit1 is an ATPase. One possibility is that the telomerase-associated Ccq1 is directly replaced at the telomere by the SHREC-bound Ccq1 to terminate elongation. In addition, telomerase dissociation might be promoted by repression of TERRA. Because the increase in TERRA expression in the *ccq1(1–441)* and *clr3*Δ mutants was mild (Supplementary Figure S11), Ccq1-Clr3 might control transcription only after telomerase action. We speculate that transcription of TERRA promotes an extendible state of the telomere end, and repression of TERRA by Ccq1-SHREC after telomere elongation might be a part of the mechanism to regulate an appropriate telomere length.

Ccq1-Clr3 might directly interact with, and sequester, Trt1 from the telomeres. To our surprise, Ccq1(130–735), which does not retain its telomerase activation ability, was able to associate with Trt1. Moreover, the Ccq1-Est1 chimera stably associated with Trt1, despite having a reduced interaction with Tpz1 and lower telomere localisation in the presence of a repressive Ccq1 complex. Our previous work revealed that Ccq1 associates with Trt1 more stably than Tpz1 (11). Thus, we have not excluded the possibility that Ccq1 can associate with Trt1 independently from Tpz1. Unlike Tpz1, Ccq1 and SHREC can localise throughout the genome (37). Therefore, it is possible that, SHREC associates with the telomerase-associated Ccq1 and moves to elsewhere on a chromosome.

Ccq1 also interacts with the Clr4 complex (45). Our genetic analyses suggest that association of Clr4 with the Ccq1 C-terminus is another possible mechanism for suppression of telomerase activity, independent of SHREC. Revealing the significance of the Clr4 complex in telomere homeostasis may therefore provide further understanding of the multifunctional protein Ccq1.

Telomerase activity is known to be blocked by destruction of Est1 (60) or by telomere localisation of the STN1-TEN1 complex (which additionally contains CTC1 in mammals) resulting in inhibition of telomerase access (32,34,61). These inhibitory mechanisms are the major pathway for termination of telomerase activity and are activated after S-phase. Our results reveal a distinct telomerase termination mechanism after telomerase recruitment, which might control the retention/processivity of telomerase. We propose that a switch between two Ccq1 complexes modulates telomerase stability at the telomere and that Ccq1-SHREC releases the Ccq1-telomerase from elongating telomeres.

Although a Ccq1 homologue has not been identified in humans, we believe that this termination mechanism may be conserved. In mammals, the core shelterin component TIN2 appears to be involved in telomerase recruitment and heterochromatin regulation. Like Ccq1 and Poz1 in fission yeast, TIN2 interacts with the C-terminus of ACD^TPP1^. Therefore, we propose that TIN2 might be a bi-functional mammalian counterpart of both Poz1 and Ccq1 (discussed in (2)). Similarly, the nucleosome remodelling and deacetylation complex (NuRD or NRD), which acts as the human counterpart to the SHREC complex (38), may have a thus far unforeseen role in telomerase regulation and telomere homeostasis. Further investigation of the relationship between chromatin remodelling factors and the telomere/telomerase may shed light on our understanding of how telomerase activity is monitored and modulated at each telomere.

## Supplementary Material

Supplementary Data
