# Supplementary material for: Fission yeast Ccq1 is a modulator of telomerase activity
Source: Nucleic Acids Res. 2017 Dec 4;46(2):704–16. doi: 10.1093/nar/gkx1223 (PMC5778466; doi:10.1093/nar/gkx1223)
Supplement: Supplementary Data [file gkx1223_supp.pdf]

Supplementary data

Supplementary Figures:

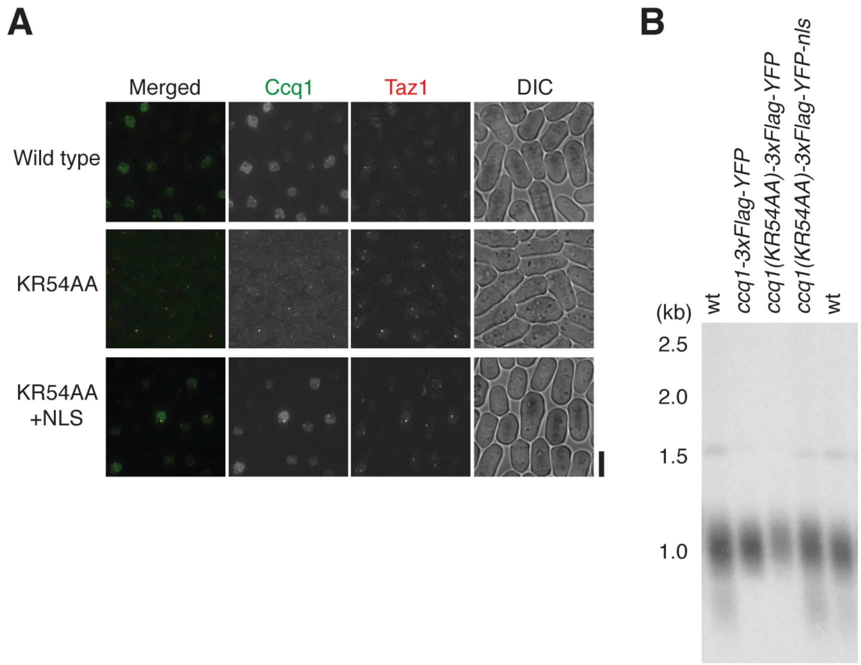

Figure S1 - Analysis of *ccq1 nls* signal peptide

A. Projected images of growing cells. Wild type and *KR54AA* mutated Ccq1 were visualised by YFP tagging. Telomeres are visualised with Taz1-mCherry. Yellow foci in the merged image indicate co-localisation of Ccq1 with telomeres (Taz1). Diffused Ccq1(KR54AA)-YFP was detected throughout the cell, and the ectopic NLS peptide fusion to Ccq1(KR54AA)-YFP rescued its nuclear localisation. Scale bar equals 10 µm.

B. Telomere Southen blot shows that the *nls* mutation (*KR54AA*) within *ccq1* or *ccq1* YFP tagging does not impair telomere length maintenance. Telomere length of the *ccq1*(*KR54AA)-nls* mutants. *Eco*RI digested telomere fragments are an average length of 1kb in wild type cells.

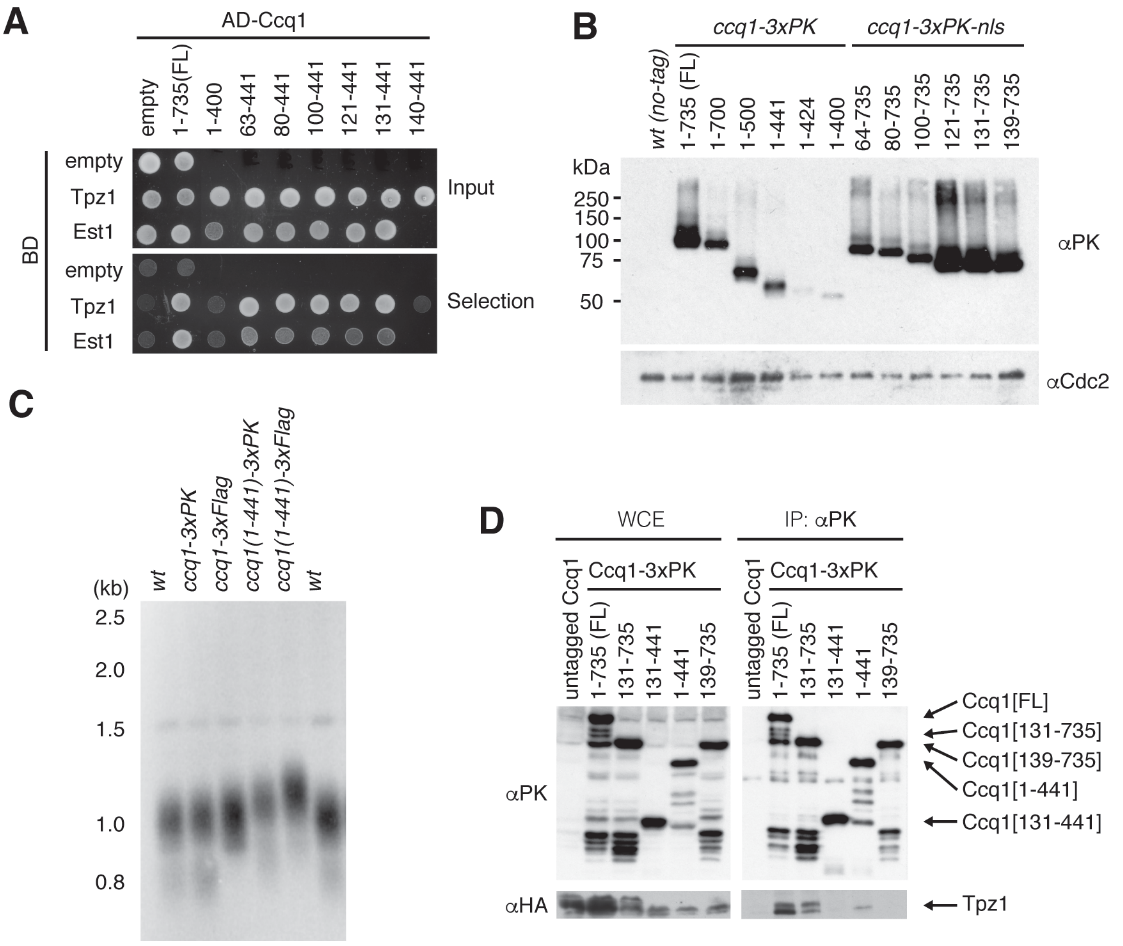

Figure S2 - Ccq1 truncation mutants

A. The yeast two-hybrid analysis for interaction of truncated Ccq1 with Tpz1 and Est1. Tpz1 interacts with amino acids 131-441 region of Ccq1. For Est1, a strong interaction was detected with full length Ccq1 but weak interaction was retained with the Tpz1 binding region of Ccq1. The indicated Ccq1 truncation proteins were fused to the GAL4 activation domain (AD) and Tpz1 and Est1 were fused to the GAL4 DNA binding domain (BD). Selection plate lacks histidine and contains 1 mM 3-AT.

B. Western blot showing expression levels of the PK-tagged Ccq1 truncations. Partial deletion of the Tpz1 binding domain, amino acids 1-424 and 1-400, impaired stability of the protein.

C. Telomere Southern blot shows that the both PK and FLAG-tagged *ccq1(1-441)* truncation mutants exhibited slight elongation of telomeres.

D. Co-immunoprecipitation of Tpz1 with Ccq1 truncations shows that the interaction is lost in Ccq1(139-735) and Ccq1(131-441).

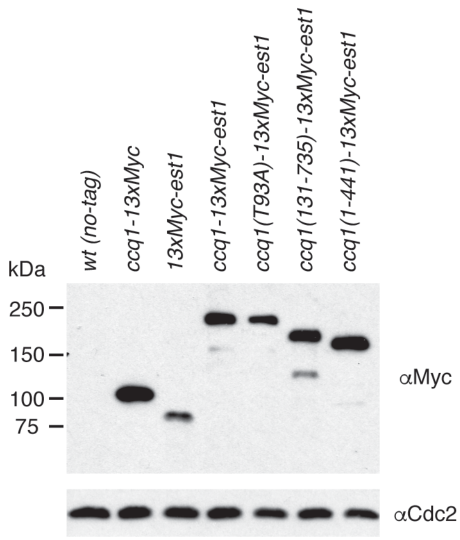

Figure S3 - Expression of the Est1-fused mutant Ccq1
Stable expression of the Ccq1-Est1 chimera proteins used in this study. Western blot showing expression levels of the Myc-tagged Ccq1 and Est1 proteins and the chimeric Ccq1-Est1 fusion product used in this study. Anti- Cdc2 was used as a control for loading of total protein.

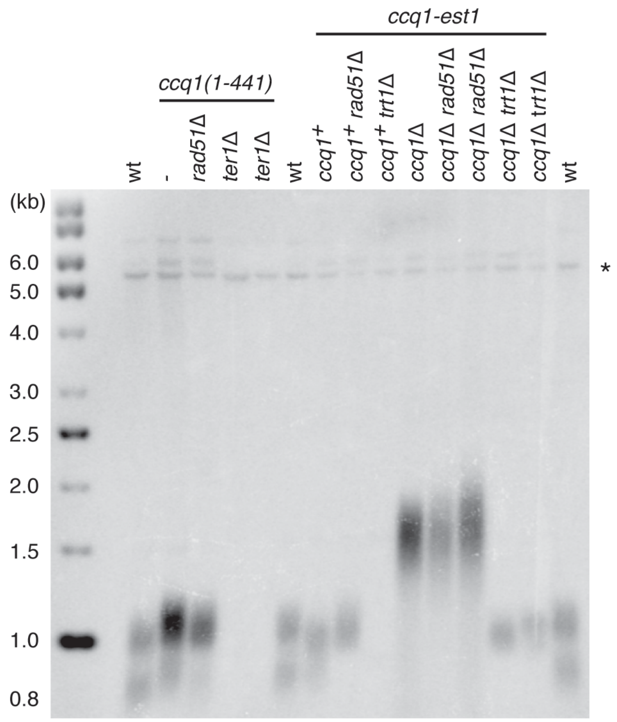

Figure S4 - Telomerase dependent telomere maintenance in *ccq1(1-441)* mutants and cells expressing the Ccq1-Est1 chimera protein
Telomere Southern blot shows that telomere length homeostasis is impaired in the absence of *TER1* or Trt1 but not Rad51 in strains expressing Ccq1(1-441) or the Ccq1-Est1 chimera. Asterisk indicates fragments containing internal telomeric DNA, used as loading control.

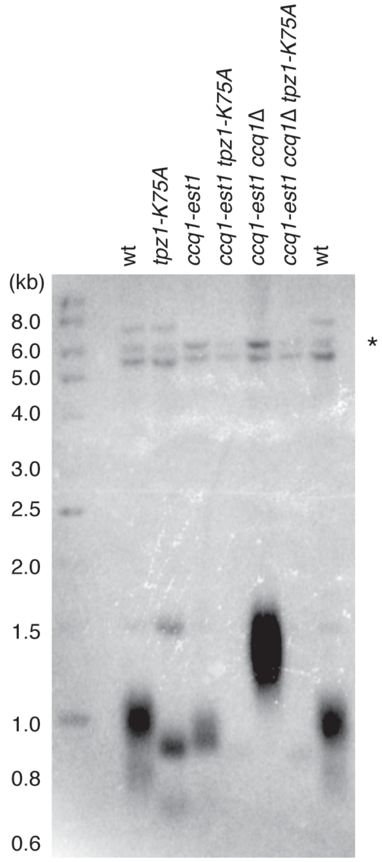

Figure S5 - Tpz1 is required for telomere maintenance

Telomere Southern blot shows that the Ccq1-Est1 chimera requires functional Tpz1-OB fold domain for telomere maintenance. The *K75A* mutation within *tpz1* causes telomerase activation defect. The *tpz1-K75A* mutation exerts a dominant telomere maintenance phenotype. Asterisk indicates fragments containing internal telomeric DNA, used as loading control.

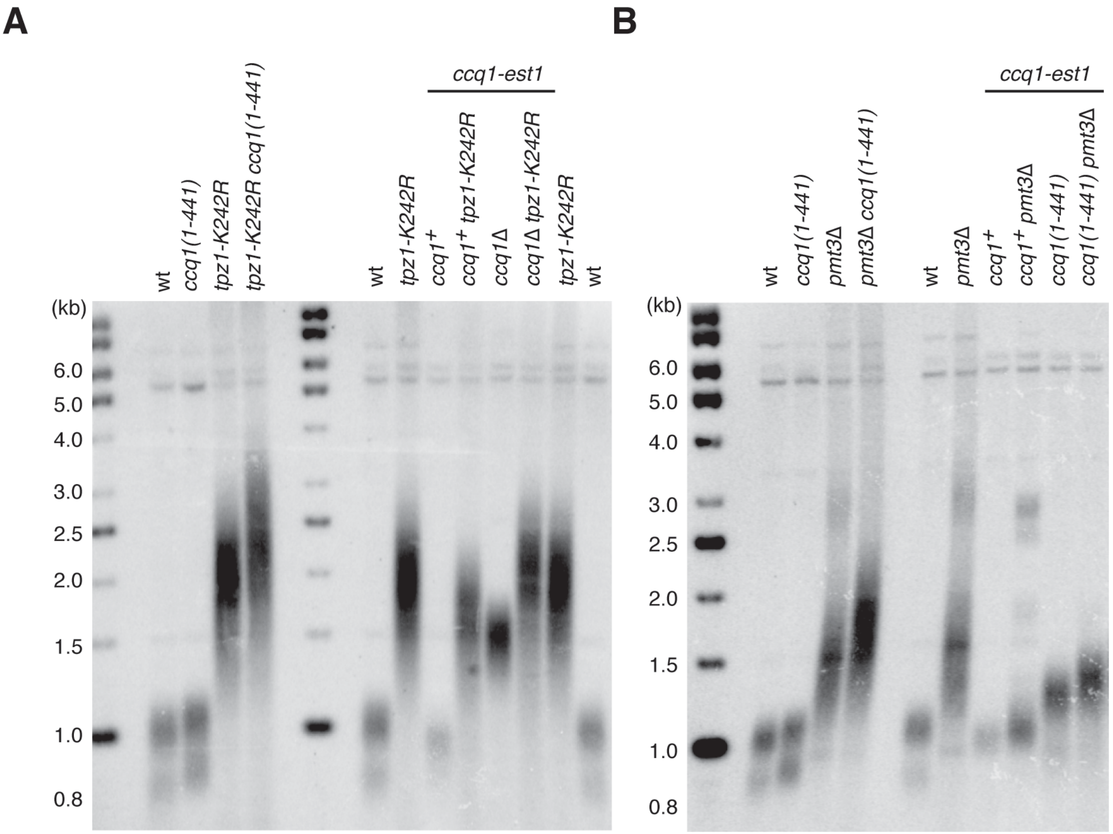

Figure S6 - Tpz1 SUMOylation is not required for negative regulation of telomere lengthening by Ccq1

Telomere Southern blot shows that additive telomere elongation of *ccq1* mutants in the absence of Tpz1 SUMOylation (A) by *tpz1-K242R* mutation and (B) by *pmt3*Δ.

A. *tpz1-K242R* mutants contain long telomeres which are further extended in the *ccq1(1-441)* and *ccq1*Δ *ccq1-est1* backgrounds. We note that expression of Ccq1-Est1 chimera slightly impairs telomerase activity, resulting in slightly shorter telomeres in *tpz1-K242R ccq1-est1*strain comparing to *tpz1-K242R* single mutant.

B. *pmt3*Δ cells contain heterogeneously elongated telomeres, which are further extended in the *ccq1(1-441)* and *ccq1(1-441) ccq1-est1* backgrounds.

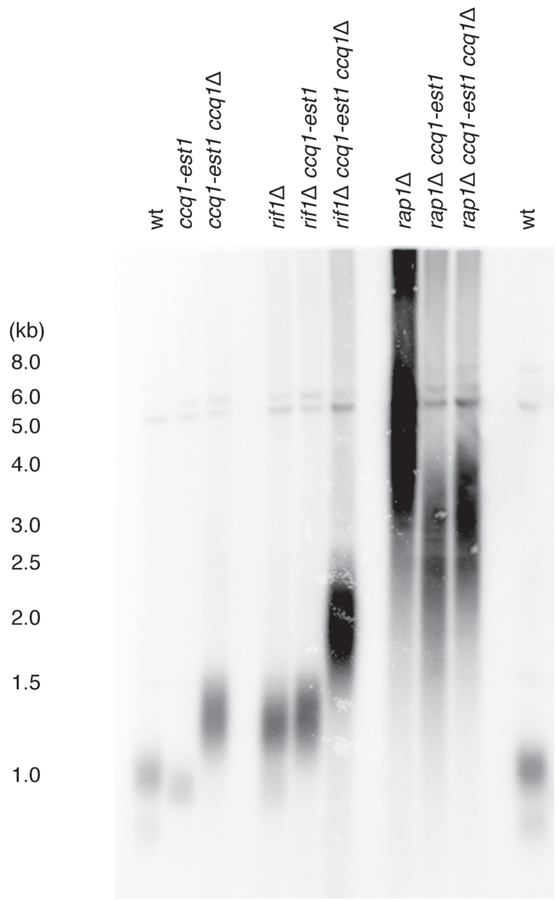

Figure S7 - *rap1* deletion leads to dominant telomere elongation over *ccq1* deletion in cells expressing Ccq1-fused Est1

Telomere Southern blot shows that *rif1* deletion in cells expressing Est1-fused Ccq1 leads to additive telomere elongation. In contrast, *rap1* deletion appears to be the dominant phenotype for telomere elongation. The efficiency of telomere elongation by *rap1*Δ was slightly reduced by the Ccq1-fusion to Est1, implying that not only association but dissociation between Ccq1 and Est1 also promotes activation of telomerase.

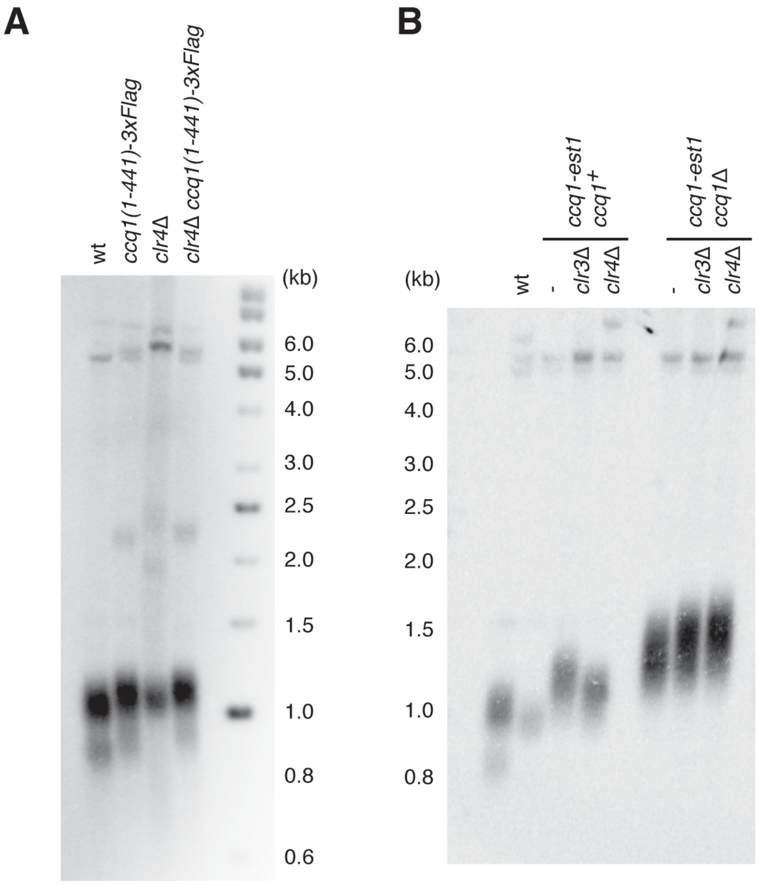

Figure S8 – Genetic interaction of *clr3* and *clr4* with *ccq1* for negative regulation of telomere length

Telomere Southern blot shows that elongation of telomeres in *clr4*Δ and *clr3*Δ is epistatic to that in *ccq1* mutants.

A. *ccq1(1-441)* and *clr4*Δ single mutants slightly elongate telomeres and are not additive in the double mutants.

B. Deletion of *clr4* and *clr3* does not cause further extension of telomeres in the *ccq1*Δ *ccq1-est1* background.

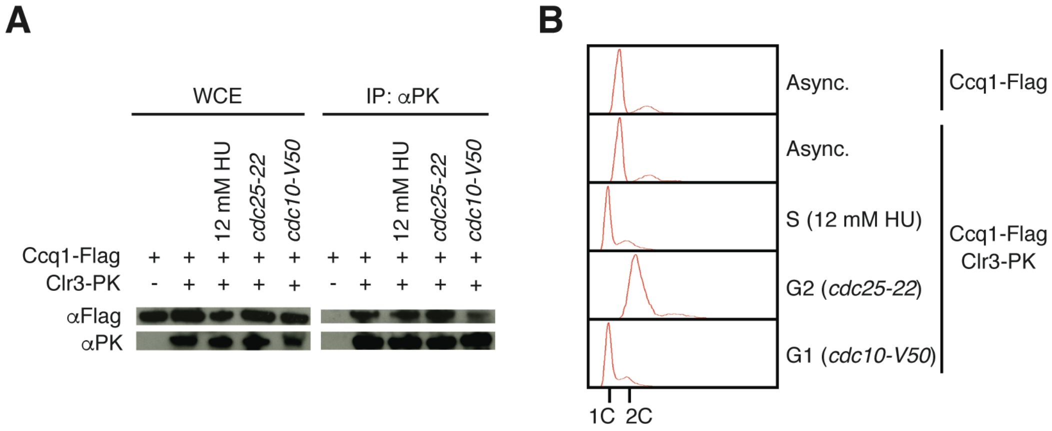

Figure S9 - Interaction of Ccq1 with Clr3 during cell cycle

Co-immunoprecipitation of Clr3-3xPK with Ccq1-Myc at the indicated cell cycle phases. Cell cycle arrest at S-, G2- and G1-phases was achieved using 12 mM HU treatment and *cdc25-22* and *cdc10-V50 ts* mutants, respectively. After 3 hours incubation at 35 °C the cells were harvested and were subjected to co-immunoprecipitation (A) and FACS (B).

A. Western blot showing interaction of Clr3 and Ccq1 at S-, G2- and G1-phases.
B. FACS analysis showing cell cycle arrest at early S-phase with HU treatment, at G2-phase by *cdc25* inactivation and at G1-phase by *cdc10* inactivation. 'Async.' corresponds to asynchronised cells. Note that *cdc25-22* dependent G2 arrest causes elongation of cells that affects FACS analysis, resulting in virtually increased DNA content.

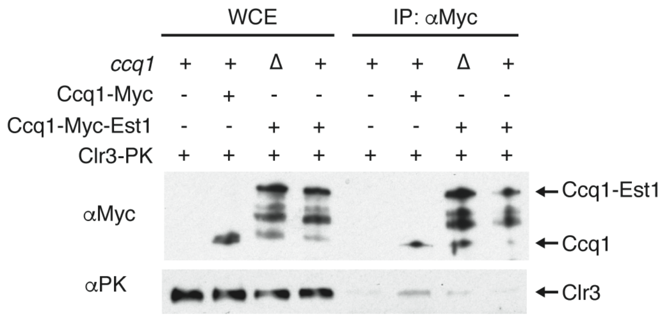

Figure S10 – Clr3 preferentially interacts with Est1-free Ccq1

Co-immunoprecipitation of Clr3-3xPK with Ccq1-Myc and the Ccq1-Myc-Est1 chimera in the presence and absence of endogenous Ccq1. Est1 fusion significantly impair interaction of Ccq1 with Clr3. Presence of endogenous Est1-free Ccq1 further reduced their interaction, presumably by outcoming interaction of endogenous Ccq1 with Clr3.

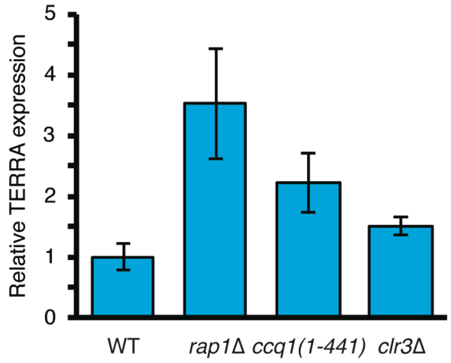

Figure S11 - Increased level of TERRA expression in *clr3*Δ and *ccq1(1-441)* truncation mutants

Graph shows relative level of endogenous telomeric transcription (TERRA) in *rap1*Δ, *ccq1(1-441)* and *clr3*Δ, compared to wild type. Expression of TERRA was determined by RT-qPCR. *rap1*Δ was used as a positive control. The small nucleolar RNA snR101 was used as an internal reference. Data are expressed as mean average from three biological replicas. p-value for significant differences (two-tailed *t*-test) over wild type indicated *p=0.017*, *0.030* and *0.051* for *rap1*Δ, *ccq1(1-441)* and *clr3*Δ, respectively.

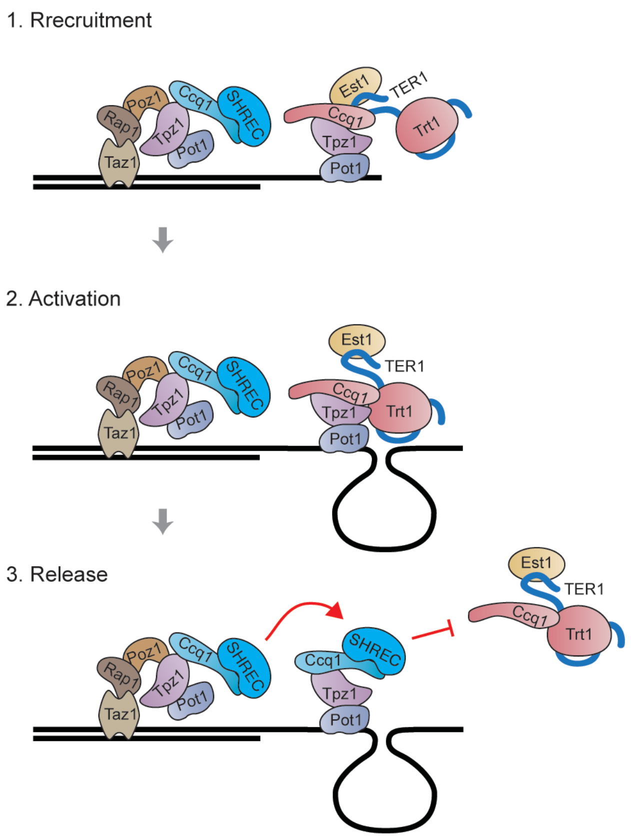

Figure S12 – Hypothetical model for telomerase release

1. Telomerase recruitment is achieved *via* Est1 interaction with Ccq1 that has been phosphorylated.
2. Telomerase add telomeric DNA with support of Ccq1 and Tpz1
3. Ccq1 C-terminus domain and SHREC within the other shelterin complex replace telomerase bound Ccq1 and release telomerase

Table S1 - Fission yeast strain list

KTP strain	Genotype
1	*h*$^{-}$
1382	*h*$^{+}$ *ccq1-3xPK:hygMX6*
1388	*h*$^{+}$ *ccq1(1-701)-3xPK:hygMX6*
1379	*h*$^{+}$ *ccq1(1-500)-3xPK:hygMX6*
1386	*h*$^{+}$ *ccq1(1-441)-3xPK:hygMX6*
1452	*h*$^{+}$ *ccq1(1-424)-3xPK:hygMX6*
1385	*h*$^{+}$ *ccq1(1-400)-3xPK:hygMX6*
306	*h*$^{+}$ *ccq1::hygMX6*
1389	*h*$^{+}$ *ccq1(63-735)-3xPK-nls:hygMX6*
1966	*h*$^{+}$ *ade6-M210 leu1-32 ura4-D18 his3-D1 ccq1(80-735)-3xPK-nls:hygMX6*
1967	*h*$^{-}$ *ade6-M210 leu1-32 ura4-D18 his3-D1 ccq1(100-735)-3xPK-nls:hygMX6*
1491	*h*$^{+}$ *ccq1(121-735)-3xPK-nls:hygMX6*
1455	*h*$^{+}$ *ccq1(131-735)-3xPK-nls:hygMX6*
1490	*h*$^{+}$ *ccq1(139-735)-3xPK-nls:hygMX6*
3427	*h*$^{+}$ *ccq1(1-441)-3xPK:hygMX6 rad51::kanMX6*

4157	h^{+} *ccq1(1-441)-3xPK:hygMX6 ter1::TKnatAX*
4158	h^{+} *ccq1(1-441)-3xPK:hygMX6 ter1::TKnatAX*
2330	h^{+} *ade6-M210 tpz1-3xHA:kanMX6 trt1-13xMyc:natMX6*
3182	h^{+} *ade6-M210 tpz1-3xHA:kanMX6 trt1-13xMyc:natMX6 ccq1-3xPK:hygMX6*
3183	h^{+} *ade6-M210 tpz1-3xHA:kanMX6 trt1-13xMyc:natMX6 ccq1(1-441)-3xPK:hygMX6*
3184	h^{+} *ade6-M210 tpz1-3xHA:kanMX6 trt1-13xMyc:natMX6 ccq1(131-735)-3xPK-nls:hygMX6*
3198	h^{+} *ade6-M210 tpz1-3xHA:kanMX6 trt1-13xMyc:natMX6 ccq1(131-441)-3xPK-nls:hygMX6*
4116	h^{-} *leu1-32 ura4-D18 tpz1-3xHA:kanMX6 trt1-13xMyc:natMX6 ccq1(139-735)-3xPK-nls:hygMX*
2109	h^{-}/h^{+} *ade6-M210/M216 leu1-32/32 ura4-D18/D18 his3-D1/D1 ccq1::ura4*$^{+}$*/+*
1446	h^{-}/h^{+} *ade6-M210/M216 leu1-32/32 ura4-D18/D18 his3-D1/D1 ccq1::ura4*$^{+}$*/+ est1::hygMX6:P*^{est1}*>ccq1-13xMyc-est1/+*
1484	h^{-} *ade6-M216 leu1-32 ura4-D18 his3-D1 est1::hygMX6:P*^{est1}*>ccq1-13xMyc-est1*
1485	h^{+} *ade6-M210 leu1-32 ura4-D18 his3-D1 est1::hygMX6:P*^{est1}*>ccq1-13xMyc-est1*
1451	h^{+} *ade6-M216 leu1-32 ura4-D18 his3-D1 ccq1::ura4*$^{+}$ *est1::hygMX6:P*^{est1}*>ccq1-13xMyc-est1*
1482	h^{+} *ade6-M216 leu1-32 ura4-D18 his3-D1 ccq1::ura4*$^{+}$ *est1::hygMX6:P*^{est1}*>ccq1-13xMyc-est1*
1486	h^{-} *ade6-M210 leu1-32 ura4-D18 his3-D1 ccq1::ura4*$^{+}$
296	h^{smt0} *leu1-32 ura4-D18 est1::hygMX6:P*^{est1}*>13xMyc-est1*
803	h^{-} *ade6-M210 ccq1-13xMyc:natMX6*
4155	*h- ura4-D18 rad51::kanMX6 est1::hygMX6:Pest1>Ccq1-13xMyc-Est1*
2537	*h- ade6-M216 leu1-32 ura4-D18 his3-D1 trt1::natMX6 est1::hygMX6:Pest1>Ccq1-13xMyc-Est1*
4153	h^{+} *ade6-M210 leu1-32 ura4-D18 rad51::ura4*$^{+}$ *ccq1::kanMX6 est1::hygMX6:Pest1>Ccq1-13xMyc-Est1*
4154	h^{+} *ade6-M210 leu1-32 ura4-D18 rad51::ura4*$^{+}$ *ccq1::kanMX6 est1::hygMX6:Pest1>Ccq1-13xMyc-Est1*
2621	h^{+} *ade6-M216 leu1-32 ura4-D18 his3-D1 trt1::natMX6 ccq1::ura4*$^{+}$ *est1::hygMX6:Pest1>Ccq1-13xMyc-Est1*
3793	h^{+} *ade6-M216 leu1-32 ura4-D18 his3-D1 ccq1::ura4*$^{+}$ *est1::hygMX6:Pest1>Ccq1-13xMyc-Est1 trt1::neoCV*
1628	h^{-} *ade6-M210 trt1-9xPK:kanMX6*
2534	h^{-} *ade6-M216 leu1-32 ura4-D18 his3-D1 trt1-9xPK:natMX6 est1::hygMX6:P*^{est1}*>ccq1-13xMyc-est1*
2563	h^{+} *ade6-M210 leu1-32 ura4-D18 his3-D1 ccq1::ura4*$^{+}$ *est1::hygMX6:P*^{est1}*>ccq1-13xMyc-est1 trt1-9xPK:natMX6*
2267	h^{+} *ccq1-3xFLAG:natMX6*
1616	h^{-} *ura4-D18 ccq1-3xFLAG:natMX6 est1::hygMX6:P*^{est1}*>ccq1-13xMyc-est1*
1556	h^{-} *ade6-M210 leu1-32 his3-D1 ccq1(1-441)-3xFLAG:natMX6 est1::hygMX6:P*^{est1}*>ccq1-13xMyc-est1*
1715	h^{+} *ade6-M216 leu1-32 ura4-D18 his3-D1 ccq1(131-735)-3xFLAG-nls:natMX6 est1::hygMX6:P*^{est1}*>ccq1-13xMyc-est1*
1723	h^{-} *ade6-M216 leu1-32 ura4-D18 his3-D1 ccq1(140-735)-3xFLAG-nls:natMX6 est1:hygMX6:P*^{est1}*>ccq1-13xMyc-est1*
1724	h^{+} *ade6-M216 leu1-32 ura4-D18 his3-D1 ccq1(500-735)-3xFLAG-nls:natMX6 est1:hygMX6:P*^{est1}*>ccq1-13xMyc-est1*
2430	h^{+} *ade6-M210 tpz1-3xHA:kanMX6 est1::hygMX6:P*^{est1}*>ccq1-13xMyc-est1*
3871	h^{+} *tpz1-3xHA:kanMX6 ccq1-3xFLAG:natMX6 est1::hygMX6:P*^{est1}*>ccq1-13xMyc-est1*
3870	h^{+} *ade6-M210 tpz1-3xHA:kanMX6 ccq1(1-441)-3xFLAG:natMX6 est1::hygMX6:P*^{est1}*>ccq1-13xMyc-est1*

3872	h^{+} ade6-M210 leu1-32 ura4-D18 tpz1-3xHA:kanMX6 ccq1(131-735)-3xFLAG-nls:natMX6 est1::hygMX6:P^{est1}>ccq1-13xMyc-est1
2431	h^{+} ade6-M210 leu1-32 ura4-D18 ccq1::ura4^{+} tpz1-3xHA:kanMX6 est1::hygMX6:P^{est1}>ccq1-13xMyc-est1
2653	h^{-} ade6-M210 leu1-32 ura4-D18 ccq1-3xFLAG:hygMX6 tpz1-3xHA:kanMX6
4025	h^{-} ccq1-3xFLAG:natMX6 est1::hygMX6:P^{est1}>13xMyc-est1
3996	h^{-} ade6-M210 leu1-32 ura4-D18 est1::hygMX6:P^{est1}>ccq1(1-441)-13xMyc-est1 ccq1-3xFLAG:natMX6 tpz1-3xHA:kanMX6
1546	h^{+}/h^{-} ade6-M210/M216 leu1-32/32 ura4-D18/D18 his3-D1/D1 ccq1::ura4^{+}/+ est1::hygMX6:P^{est1}>ccq1(1-441)-13xMyc-est1/+
1624	h^{+} ade6-M210 leu1-32 ura4-D18 his3-D1 est1::hygMX6:P^{est1}>ccq1(1-441)-13xMyc-est1
1625	h^{+} ade6-M216 leu1-32 ura4-D18 his3-D1 ccq1::ura4^{+} est1::hygMX6:P^{est1}>ccq1(1-441)-13xMyc-est1
3571	h^{+} tpz1-3xHA:kanMX6 clr3-3xPK:hygMX6
3689	h^{+} ade6-M210 ura4-D18 ccq1-3xFLAG:hygMX6 tpz1-3xHA:kanMX6 clr3-3xPK:hygMX6
3616	h^{+} ura4-D18 ccq1(1-441)-3xFLAG:natMX6 tpz1-3xHA:kanMX6 clr3-3xPK:hygMX6
3622	h^{+} leu1-32 ura4-D18 ccq1(500-735)-3xFLAG-nls:natMX6 tpz1-3xHA:kanMX6 clr3-3xPK:hygMX6
1732	h^{+} ccq1(1-441)-3xFLAG:natMX6
732	h^{-} ade6-M210 leu1-32 ura4-D18 his3-D1 clr3::natMX6
3931	h^{+} leu1-32 ura4-D18 mit1::zeoCV
576	h^{-} leu1-32 ura4-D18 his3-D1 lys1-131 clr4::kanMX6
704	h^{-} ade6-M216 leu1-32 his3-D1 swi6::kanMX6
3893	h^{-} ade6-M216 leu1-32 ura4-D18 his-D1 clr3::natMX6 est1::hygMX6:P^{est1}>ccq1-13xMyc-est1
3964	h^{+} mit1::zeoCV est1::hygMX6:P^{est1}> ccq1-13xMyc-est1
3890	h^{+} leu1-32 ura4-D18 his3-D1 clr4::kanMX6 est1::hygMX6:P^{est1}>ccq1-13xMyc-est1
3897	h^{-} ade6-M210 leu1-32 swi6::kanMX6 est1::hygMX6:P^{est1}>ccq1-13xMyc-est1
3935	h^{-} ura4-D18 clr3-3xPK:hygMX6
3962	h^{-} ura4-D18 clr3-3xPK:kanMX6 ccq1-13xMyc:natMX6
3963	h^{+} leu1-32 ura4-D18 his3-D1 ccq1::ura4^{+} est1::hygMX6:P^{est1}>ccq1-13xMyc-est1 clr3-3xPK:kanMX6
3946	h^{+} ade6-M216 leu1-32 ura4-D18 his-D1 est1::hygMX6:Pest1>Ccq1-13xMyc-Est1 clr3-3xPK:hygMX6
236	h^{90} ade6-M210 leu1-32 ura4-D18 his3-D1 otr1R:ade6^{+} telo1L:his3^{+} telo2L:ura4^{+}
238	h^{90} ade6-M210 leu1-32 ura4-D18 his3-D1 otr1R:ade6^{+} telo1L:his3^{+} telo2L:ura4^{+} taz1::hygMX6
3908	h^{90} ade6-M210 leu1-32 ura4-D18 his3-D1 otr1R:ade6^{+} telo2L:ura4^{+} ccq1::hygMX6
3883	h^{90} ade6-M210 leu1-32 ura4-D18 his3-D1 otr1R:ade6^{+} telo1L:his3^{+} telo2L:ura4^{+} ccq1(1-441)-3xPK:hygMX6
3934	h^{90} ade6-M210 leu1-32 ura4-D18 his3-D1 otr1R:ade6^{+} telo2L:ura4^{+} ccq1::natCX est1::hygMX6:P^{est1}>ccq1-13xMyc-est1
3892	h^{90} ade6-M210 leu1-32 ura4-D18 his3-D1 otr1R:ade6^{+} telo1L:his3^{+} telo2L:ura4^{+} clr3::natMX6
237	h^{90} ade6-M210 leu1-32 ura4-D18 his3-D1 otr1R:ade6^{+} telo1L:his3^{+} telo2L:ura4^{+} clr4::kanMX6
602	h^{+} leu1-32 ura4-D18 his3-D1 rap1::natMX6
2244	h^{+} ura4-D18 taz1-mCherry:kanMX6 ccq1-3xFLAG-YFP:natMX6
2247	h^{+} ura4-D18 taz1-mCherry:kanMX6 ccq1(KR54AA)-3xFLAG-YFP:natMX6
1611	h^{+} ura4-D18 taz1-mCherry:kanMX6 ccq1(KR54AA)-3xFLAG-YFP-nls:natMX6

797	h^{+} ura4-D18 ccq1-3xFLAG-YFP:natMX6
796	h^{+} ura4-D18 ccq1(KR54AA)-3xFLAG-YFP:natMX6
2266	h^{+} ccq1-KR54AA-3xFLAG-YFP-nls:natMX6
1466	h^{-}/h^{+} ade6-M210/M216 ccq1(T93A)-3xflag:natMX6/+
1889	h^{-} ade6-M216 ccq1(T93A)-3xFLAG:natMX6
1502	h^{-}/h^{+} ade6-M210/M216 leu1-32/32 ura4-D18/D18 his3-D1/D1 ccq1::$ura4^{+}$/+ est1::hygMX6:P^{est1}>ccq1(T93A)-13xMyc-est1/+
1516	h^{-} ade6-M210 leu1-32 ura4-D18 his3-D1 est1::hygMX6:P^{est1}>ccq1(T93A)-13xMyc-est1
1517	h^{-} ade6-M216 leu1-32 ura4-D18 his3-D1 ccq1::$ura4^{+}$ est1::hygMX6:P^{est1}>ccq1(T93A)-13xMyc-est1
1501	h^{-}/h^{+} ade6-M210/M216 leu1-32/32 ura4-D18/D18 his3-D1/D1 ccq1::$ura4^{+}$/+ est1::hygMX6:P^{est1}>ccq1(131-735)-13xMyc-est1/+
1512	h^{-} ade6-M210 leu1-32 ura4-D18 his3-D1 est1::hygMX6:P^{est1}>ccq1(131-735)-13xMyc-est1
1510	h^{-} ade6-M216 leu1-32 ura4-D18 his3-D1 ccq1::$ura4^{+}$ est1::hygMX6:P^{est1}>ccq1(131-735)-13xMyc-est1
2360	h^{+} ade6-M210 tpz1(K75A)-3xHA:kanMX6
2417	h^{+} ade6-M210 ura4-D18 tpz1(K75A)-3xHA:kanMX6 est1::hygMX6:P^{est1}>ccq1-13xMyc-est1
3794	h^{+} ade6-M216 leu1-32 ura4-D18 his3-D1 ccq1::$ura4^{+}$ est1::hygMX6:P^{est1}>ccq1-13xMyc-est1 tpz1(K75A)-3xHA:kanMX6
3788	h^{+} ade6-M210 tpz1(K242R)-3xHA:kanMX6
4152	h^{+} tpz1(K242R)-3xHA:kanMX6 ccq1(1-441)-3xPK:hygMX6
4124	h- ade6-M216 leu1-32 ura4-D18 his3-D1 tpz1(K242R)-3xHA:kanMX6 est1::hygMX6:Pest1>Ccq1-13xMyc-Est1
4138	h^{+} ade6-M216 leu1-32 ura4-D18 his3-D1 ccq1::$ura4^{+}$ tpz1(K242R)-3xHA:kanMX6 est1::hygMX6:Pest1>Ccq1-13xMyc-Est1
733	h^{-} leu1-32 ura4-D18 pmt3::$ura4^{+}$
3424	h^{+} leu1-32 ura4-D18 pmt3::$ura4^{+}$ ccq1(1-441)-3xPK:hygMX6
4119	h^{-} ade6-M210 leu1-32 ura4-D18 pmt3::$ura4^{+}$ est1::hygMX6:Pest1>Ccq1-13xMyc-Est1
4135	h^{+} leu1-32 ura4-D18 his3-D1 pmt3::$ura4^{+}$ ccq1(1-441)-3xflag:natMX6 est1::hygMX6:Pest1>Ccq1-13xMyc-Est1
2001	h^{-} ade6-M210 leu1-32 ura4-D18 his3-D1 rif1::kanMX6
1549	h^{+} ade6-M210 leu1-32 ura4-D18 his3-D1 rif1::kanMX6 est1::hygMX6:P^{est1}>ccq1-13xMyc-est1
1547	h^{-} ade6-M216 leu1-32 ura4-D18 his3-D1 rif1::kanMX6 ccq1::$ura4^{+}$ est1::hygMX6:P^{est1}>ccq1-13xMyc-est1
2420	h^{+} ade6-M216 leu1-32 his3-D1 rap1::natMX6 est1::hygMX6:P^{est1}>ccq1-13xMyc-est1
2406	h^{+} leu1-32 ura4-D18 his3-D1 rap1::natMX6 ccq1::$ura4^{+}$ est1::hygMX6:P^{est1}>ccq1-13xMyc-est1
3990	h^{-} ade6-M210 ura4-D18 clr4::kanMX6 ccq1(1-441)-3xflag:natMX6
3869	h^{+} ade6-M216 leu1-32 ura4-D18 his-D1 clr3::natMX6 ccq1::$ura4^{+}$ est1::hygMX6:Pest1>Ccq1-13xMyc-Est1
3889	h^{+} ade6-M216 leu1-32 ura4-D18 his3-D1 clr4::kanMX6 ccq1::$ura4^{+}$ est1::hygMX6:Pest1>Ccq1-13xMyc-Est1
3869	h^{+} ade6-M216 leu1-32 ura4-D18 his-D1 clr3::natMX6 ccq1::$ura4^{+}$ est1::hygMX6:Pest1>Ccq1-13xMyc-Est1
4182	h^{-} ade6-704 leu1-32 ura4-D18 cdc25-22 clr3-3xPK:hygMX6 ccq1-3xflag:natMX6
4181	h^{+} ade6-M210 ura4-D18 cdc10-V50 clr3-3xPK:hygMX6 ccq1-3xflag:natMX6

Use of "*est1::hygMX6:*P^{est1}> " before a gene indicates that *hygMX6* marker is inserted upstream of the promoter and the corresponding gene is expressed from the *est1* promoter at endogenous *est1* locus.
